# IRF4 Mediates the Oncogenic Effects of STAT3 in Anaplastic Large Cell Lymphomas

**DOI:** 10.3390/cancers10010021

**Published:** 2018-01-18

**Authors:** Cecilia Bandini, Aldi Pupuleku, Elisa Spaccarotella, Elisa Pellegrino, Rui Wang, Nicoletta Vitale, Carlotta Duval, Daniela Cantarella, Andrea Rinaldi, Paolo Provero, Ferdinando Di Cunto, Enzo Medico, Francesco Bertoni, Giorgio Inghirami, Roberto Piva

**Affiliations:** 1 Department of Molecular Biotechnology and Health Sciences, University of Torino, Torino 10126, Italy; cecilia.bandini@edu.unito.it (C.B.); aldi.pupuleku@upf.edu (A.P.); elisa.spaccarotella@med.uniupo.it (E.S.); elisa.pellegrino@unito.it (E.P.); nicoletta.vitale@unito.it (N.V.); karloz.duval@hotmail.it (C.D.); paolo.provero@unito.it (P.P.); ferdinando.dicunto@unito.it (F.D.C.); ggi9001@med.cornell.edu (G.I.); 2 Center for Experimental Research and Medical Studies (CeRMS), University of Torino, Torino 10126, Italy; 3 Department of Oncology and Hemato-Oncology, University of Milan, Milan 20122, Italy; 4 Department of Experimental and Health Sciences, University Pompeu Fabra, Barcelona 08003, Spain; 5 Division of Hematology, Department of Translational Medicine, University of Eastern Piedmont, Novara 28100, Italy; 6 Department of Pathology and Laboratory Medicine, Weill Cornell Medical College, New York, NY 10065, USA; ruw2015@med.cornell.edu; 7 Candiolo Cancer Institute, FPO-IRCCS, Candiolo 10060, Italy; daniela.cantarella@ircc.it (D.C.); enzo.medico@ircc.it (E.M.); 8 Lymphoma and Genomics Research Program, IOR Institute of Oncology Research, Bellinzona 6500, Switzerland; andrea.rinaldi@ior.iosi.ch (A.R.); frbertoni@mac.com (F.B.); 9 Center for Translational Genomics and Bioinformatics, San Raffaele Scientific Institute, Milan 20132, Italy; 10 Neuroscience Institute Cavalieri Ottolenghi, University of Turin, Turin 10043, Italy; 11 Molecular Biotechnology Center, University of Torino, Torino 10126, Italy; 12 Department of Oncology, University of Torino, Candiolo 10060, Italy

**Keywords:** anaplastic large cell lymphomas, ALK, STAT3, IRF4, immunomodulatory drugs, JQ1

## Abstract

Systemic anaplastic large cell lymphomas (ALCL) are a category of T-cell non-Hodgkin’s lymphomas which can be divided into anaplastic lymphoma kinase (ALK) positive and ALK negative subgroups, based on ALK gene rearrangements. Among several pathways aberrantly activated in ALCL, the constitutive activation of signal transducer and activator of transcription 3 (STAT3) is shared by all ALK positive ALCL and has been detected in a subgroup of ALK negative ALCL. To discover essential mediators of STAT3 oncogenic activity that may represent feasible targets for ALCL therapies, we combined gene expression profiling analysis and RNA interference functional approaches. A shRNA screening of STAT3-modulated genes identified interferon regulatory factor 4 (IRF4) as a key driver of ALCL cell survival. Accordingly, ectopic IRF4 expression partially rescued STAT3 knock-down effects. Treatment with immunomodulatory drugs (IMiDs) induced IRF4 down regulation and resulted in cell death, a phenotype rescued by IRF4 overexpression. However, the majority of ALCL cell lines were poorly responsive to IMiDs treatment. Combination with JQ1, a bromodomain and extra-terminal (BET) family antagonist known to inhibit MYC and IRF4, increased sensitivity to IMiDs. Overall, these results show that IRF4 is involved in STAT3-oncogenic signaling and its inhibition provides alternative avenues for the design of novel/combination therapies of ALCL.

## 1. Introduction

Systemic anaplastic large cell lymphomas (ALCL) are a rare type of T-cell lymphoma comprising a histologically heterogeneous group of hematopoietic neoplasms, often characterized by large cells with variable shape (anaplastic pattern), consistently expressing the CD30 antigen [1]. ALCL accounts for approximately 10–15% of pediatric/adolescent non-Hodgkin lymphomas (NHL) and only 2% of adult NHL [2]. The current WHO classification of lymphoid neoplasms further subdivides systemic ALCL into anaplastic lymphoma kinase ALK-positive (6.6%) and ALK-negative (5.5%), according to ALK protein expression in tumor samples [3]. Whereas the oncogenic activity of chimeric ALK proteins is considered causative of ALK-positive ALCL, the pathogenesis of ALK-negative ALCL remains less clarified, despite recent progresses [4,5,6,7,8,9]. ALK is a membrane associated tyrosine kinase involved in the development and function of the nervous system, where it controls cell proliferation, survival, and differentiation in response to extracellular stimuli [4,5,6]. Since the discovery of ALK translocations in ALK-positive ALCL, a variety of mechanisms leading to aberrant/ectopic ALK signaling in several human cancers have been characterized. These include translocations or structural rearrangements, mutations, gene amplification, and alternative transcription start sites [7,8]. The most common alteration in ALK-positive ALCL is the t(2;5)(p23;q35) translocation that leads to the expression of nucleophosmin (NPM)-ALK chimeric protein [9,10]. 

Numerous studies have demonstrated the role of ALK fusion proteins in promoting lymphomagenesis [11,12,13], by constitutively activating various downstream signaling pathways, such as janus kinase (JAK)/signal transducer and activator of transcription (STAT), phosphatidylinositol-3-kinase (PI3K)/AKT/mammalian target of rapamycin (mTOR), RAS–extracellular signal regulated kinase (ERK), and others [6,14,15]. STAT3 is a member of the STAT protein family. Upon cytokines and growth factors activation, STAT family members are phosphorylated by the receptor associated kinases, and then form homo- or heterodimers that translocate to the cell nucleus where they act as transcription activators. In ALK-positive ALCL, it has been widely demonstrated that the oncogenic effects of ALK chimeras are mostly mediated by STAT3 [15,16,17,18,19]. Gain-of-function JAK1 and STAT3 mutations have been reported in a significant proportion (∼20%) of ALK negative ALCL [20]. These mutations were usually associated with increased phosphorylation of protein and enhanced growth activity. Interestingly, STAT3 activation has been detected in ∼47% of ALK negative ALCL [21], and JAK inhibitor sensitivity was correlated with the STAT3 phosphorylation status independently of JAK1/STAT3 mutations [22]. The pleiotropic effects of STAT3 are due to the concomitant activation/repression of multiple sets of genes, which control crucial functions, such as cell cycle, apoptosis, motility, immune response, metabolic pathways, angiogenesis and others [12,15,19,23,24,25]. In addition, STAT3 controls the expression of epigenetic modulators and numerous microRNAs [26,27,28]. The strict requirement for STAT3 in a large fraction of ALCL makes this molecule an attractive therapeutic target. However, targeting STAT3 has proven challenging and so other potential targets in this pathway have gathered attention [29]. 

Here, to discover critical mediators of STAT3 oncogenic activity that may represent viable targets for ALCL therapies, we combined gene expression profiling analysis with RNA interference functional approaches. Transcriptional analysis identified a selected number of genes (1730) specifically modulated by STAT3 silencing, which were grouped in 12 clusters, according to the kinetic and direction of their modulation. Among early-regulated genes carrying conserved STAT3 binding sites (BS) in their regulatory regions, we found that Interferon Regulatory Factor 4 (IRF4) is a key protein involved in ALCL proliferation and survival. Overall, these findings show that IRF4 is involved in STAT3-oncogenic signaling, and that its inhibition might represent a promising avenue for the design of combination therapies in ALCL. 

## 2. Results

### 2.1. Kinetics of STAT3-Regulated Genes in ALCL Cell Lines

**Figure 1:**
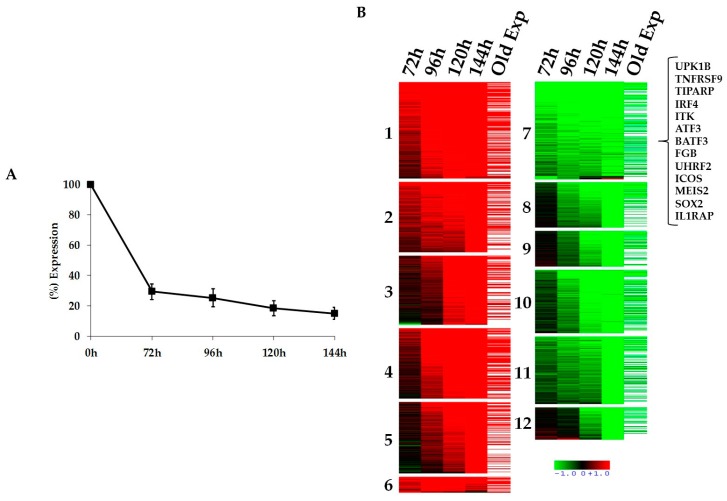
Kinetics of signal transducer and activator of transcription 3 (STAT3)-regulated genes in anaplastic large cell lymphomas (ALCL). (**A**) RT-qPCR analysis shows progressive decrease of STAT3 mRNA levels in the anaplastic lymphoma kinase (ALK) positive cell line TS-SUP-M2 S3S after doxycycline treatment (1 µg/mL). Pellet were collected at 72, 96, 120, 144 h. Error bars represent the standard deviation (s.d.) of triplicate measurements. (**B**) Heatmap representation of gene expression profile analysis after STAT3 inducible knockdown in the ALK positive cell line TS-SUP-M2 S3S. Biological triplicate were used for each experimental condition. Hybridization was carried out on HumanHT-12 v4.0 Expression BeadChip (Illumina Inc., San Diego, CA, USA). STAT3 modulated genes were grouped in 12 clusters. Upregulated RNAs are shown in red, downregulated RNA are shown in green. The colour bar represents relative gene expression changes. In brackets are shown genes selected for functional validation.

To unravel the STAT3 signaling network in T-cell lymphomas and to discover key players that may represent feasible targets for ALCL therapies, we designed a time course gene expression profiling experiment (GEP) using two clones (2X and 21) of the ALK positive ALCL cell line (TS-SUP-M2 S3S) which express a doxycycline-inducible STAT3 short hairpin RNA (shRNA) [27,30]. Biological triplicates of TS-SUP-M2 S3S cells were treated with doxycycline (1 µg/mL) for 0, 24, 48, 72, 96 h. Cells were harvested for RNA and protein extraction at different time points (Figure S1A). STAT3 silencing was confirmed by RT-qPCR (Figure 1A) and by western blotting (Figure S1B). To define differential expressed genes, we used a filtering criteria of at least 2.0-Fold Change in expression (FC > 2) with a differential score of *p* < 0.001. The top hits included transcripts previously described to be regulated by NPM-ALK through STAT3, such as IL2RA, LEF1, ICOS, IL10, GAS1, SGK, and others (Table S1) [30,31]. Gene expression profiling analysis identified a selected number of genes (1730) specifically modulated by STAT3 silencing, which could be grouped in 12 clusters according to the kinetic and direction of their modulation (Figure 1B). Kinetic analysis indicates a progressive increase of differentially regulated genes (Figure S1C). The list of differentially expressed genes was further compared with a previous study in which STAT3 expression was abrogated by 3 different shRNAs [30]. Overall, more than 50% of the genes overlapped between the two experiments (Figure S1D). We then analyzed the 12 clusters for enrichment in genes carrying conserved STAT3 binding sites (BS) in their regulatory regions (Figure S2A). 

To this end, we exploited a positional weight matrix (PWM) approach, as previously described [32]. As a result, Cluster 7 (early down-regulated genes) showed a strong over-representation of putative STAT3 BS. This cluster includes 82 genes bearing one BS and 54 genes carrying two STAT3 BS, with a p value of 0.00082 and 0.00043, respectively (Figure S2B).

### 2.2. Functional Validation of STAT3 Target Genes

Many genes transcriptionally regulated by STAT3 in ALCL cells display unknown functions or have never been related to oncogenic activities in T cells. Therefore, to dissect the signaling cascade mediated by STAT3, we undertook a functional screening by RNA interference focusing on cluster 7, which includes early regulated genes, enriched for STAT3 BS. Based on literature searches and predicted functions, 13 genes of cluster 7 were selected as candidates expected to promote proliferation and/or survival of ALCL cells, via a direct STAT3 regulation (Table S2). We systematically analyzed their biological functions by a lentiviral shRNA screening employing at least 5 shRNA sequences for each target gene. To recognize genes actively driving the oncogenic properties of STAT3 in ALCL cells, we monitored biological effects of each gene knockdown using as readouts cell proliferation, survival and morphology. Positive hits were selected according to the following criteria: more than one shRNA sequence was needed to reduce target mRNA levels by at least 70%; a correlation between the proportion of gene silencing and the phenotype was required. IL-2 inducible T-cell Kinase (ITK) and Interferon regulatory factor 4 (IRF4) genes fulfilled both criteria showing a strong phenotype related to the silencing effects of three independent shRNAs (Table S3). 

### 2.3. IRF4 is Required for Proliferation and Survival of ALCL Cells

**Figure 2:**
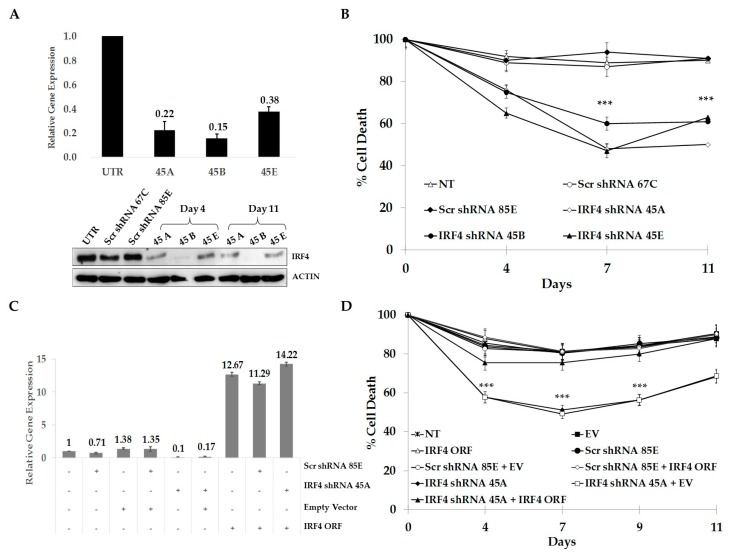
Interferon regulatory factor 4 (IRF4) is required for proliferation and survival of ALCL cells. (**A**) TS-SUP-M2 S3S cells were transduced with lentiviral particles expressing three shRNA (45A, 45B, 45E) targeting IRF4. IRF4 silencing was monitored by RT-qPCR 96 h post transduction (upper panel) and by immunoblotting at the indicated time points (bottom panel). (**B**) Viability of TS-SUP-M2 S3S cells transduced with the indicated shRNAs was monitored by tetrametylrodamine methyl ester (TMRM) staining-flow cytometry at different time points. (**C**) TS-SUP-M2 S3S cells were transduced with human IRF4 open reading frame (ORF) and empty vector as a control, selected by blasticidin (5 µg/mL), infected with a shRNA (45A) targeting IRF4 5′UTR or a control shRNA (85E), then selected with puromycin (1 mg/mL). Endogenous and ectopic IRF4 levels were detected by RT-qPCR 96 h post-infection. (**D**) Viability of TS-SUP-M2 S3S cells transduced with the indicated constructs was monitored by TMRM staining-flow cytometry at different time points. Error bars represent the s.d. of triplicate measurements (*** *p* < 0.001).

To study more in detail the role of IRF4 in ALCL cells, three shRNA sequences (45A, 45B, 45E) directed against human IRF4 were individually transduced into TS-SUP-M2 S3S cells. RT-qPCR and immunoblotting analyses revealed that IRF4 mRNA and protein levels significantly decreased in cells transduced with IRF4-directed shRNA, as compared to untransduced cells or cells transduced with control shRNA sequences (Figure 2A). A time course experiment revealed that IRF4 depletion significantly affected cell survival, as compared to controls (Figure 2B). To demonstrate the specificity of gene knock-down, we showed reversion of toxicity by ectopic expression of IRF4 or by concomitant use of a shRNA targeting the untranslated region of the respective mRNA (Figure 2C,D). On the contrary, expression of a shRNA-resistant ITK failed to rescue cells from apoptosis (data not shown). Together, these results demonstrate the requirement of IRF4 for growth and survival of ALCL cells and suggest that shRNA “off target” effects might be responsible for the cell death-induced by ITK knock-down [33].

### 2.4. IRF4 Partially Mediates STAT3 Oncogenic Properties in ALCL Cells

**Figure 3:**
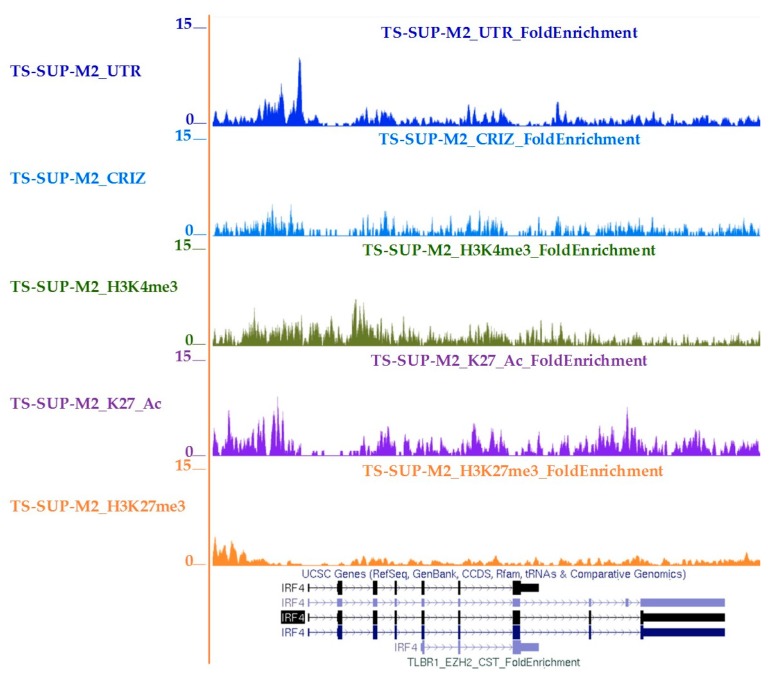
STAT3 binds to IRF4 regulatory regions in ALK-positive ALCL cells. STAT3 (blue), H3K4me (green), H3K27Ac (purple) and H3K27me3 (orange) bindings to IRF4 in TS-SUP-M2 cells. Loss of STAT3 binding (light blue) was achieved after crizotinib treatment (6 h, 200 nM). *y*-axis values represent read densities normalized to total number of reads.

**Figure 4:**
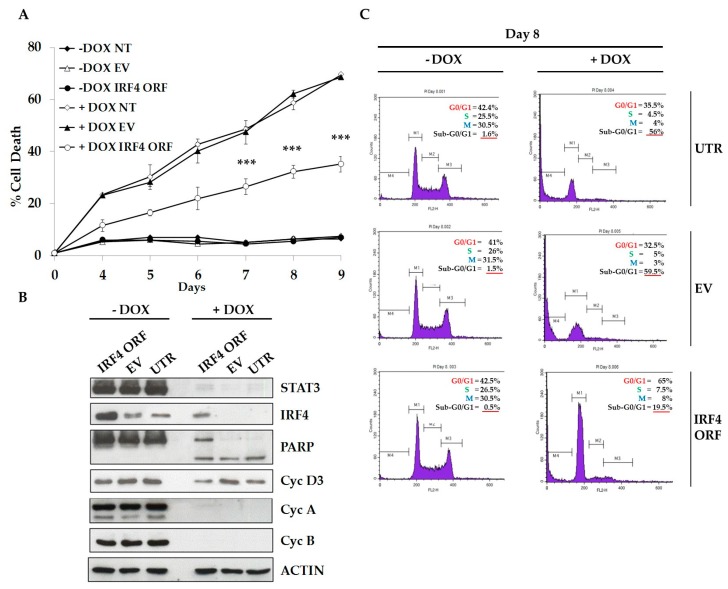
IRF4 partially mediates STAT3 oncogenic properties in ALCL cells. (**A**) TS-SUP-M2 S3S cells were transduced with lentiviral particles expressing human IRF4 open reading frame (ORF), an empty vector (EV), or left untransduced (UTR) as negative controls. Cells were cultured in the presence of 1 µg/mL doxycycline to induce STAT3 KD. Kinetics of cell death induced by conditional STAT3 KD revealed that cells expressing IRF4 displayed lower apoptotic rates compared to controls. Apoptosis analysis was performed by TMRM staining-flow cytometry at the indicated time points after doxycycline treatment. Error bars represent the s.d. of triplicate measurements (*** *p* < 0.001). (**B**) Western blot analysis of the experiment described above revealing that IRF4 over-expressing cells display lower levels of processed PARP, and invariant levels of cyclin A, B1 and D3 following STAT3 KD as compared to control cells. (**C**) Propidium iodide staining analysis of the experiment described above indicating that IRF4 over-expressing cells display reduced sub-G0/G1 fraction, indicative of decreased apoptosis. These findings are representative of three independent experiments.

Since gene expression profiling analysis indicated that IRF4 was positively regulated by STAT3, we confirmed that IRF4 mRNA and protein levels were accordingly modulated following STAT3 KD (Figure S3). STAT3 chromatin immunoprecipitation-sequencing (ChIP-Seq) analysis demonstrated that STAT3 physically interacts to IRF4 regulatory regions. STAT3 binding was completely abrogated by Crizotinib, a small-molecule inhibitor of ALK tyrosine kinase and downstream signaling mediators such as STAT3, thus confirming specific regulation of IRF4 by STAT3 (Figure 3). We then asked whether IRF4 is an essential mediator of STAT3 oncogenic activities in ALCL cells. To test this hypothesis, we forced IRF4 expression and monitored cell cycle and apoptosis after inducible STAT3 KD. We observed that TS-SUP-M2 S3S cells transduced with human IRF4 exhibited a significant lower rate of apoptosis following STAT3 down-regulation, as compared to empty vector (EV) or untransduced cells (Figure 4A). STAT3 silencing and IRF4 over-expression were confirmed by western blotting, at day 9 after doxycycline treatment (Figure 4B). To elucidate IRF4-mediated cell death prevention, we analyzed proteins involved in cell cycle and apoptosis. As shown in Figure 3B, no significant changes in cyclin D3, cyclin A, and cyclin B1 protein levels could be detected. On the contrary, ectopic IRF4 expression decreased cleavage of the caspases substrate poly (ADP ribose) polymerase 1 (PARP1), as compared to control. Accordingly, propidium iodide (PI) staining identified a lower proportion of hypodiploid cells, indicative of apoptosis, in IRF4 expressing cells (19.5%), as compared to untransduced (56%), and to empty vector (59.5%) conditions (Figure 3C). Overall, these experiments indicate that IRF4 is involved in cellular process essential for the survival of ALK positive ALCL cells. 

### 2.5. ALCL Cell Lines Display Heterogeneous Sensitivity to Immunomodulatory Drugs

**Figure 5:**
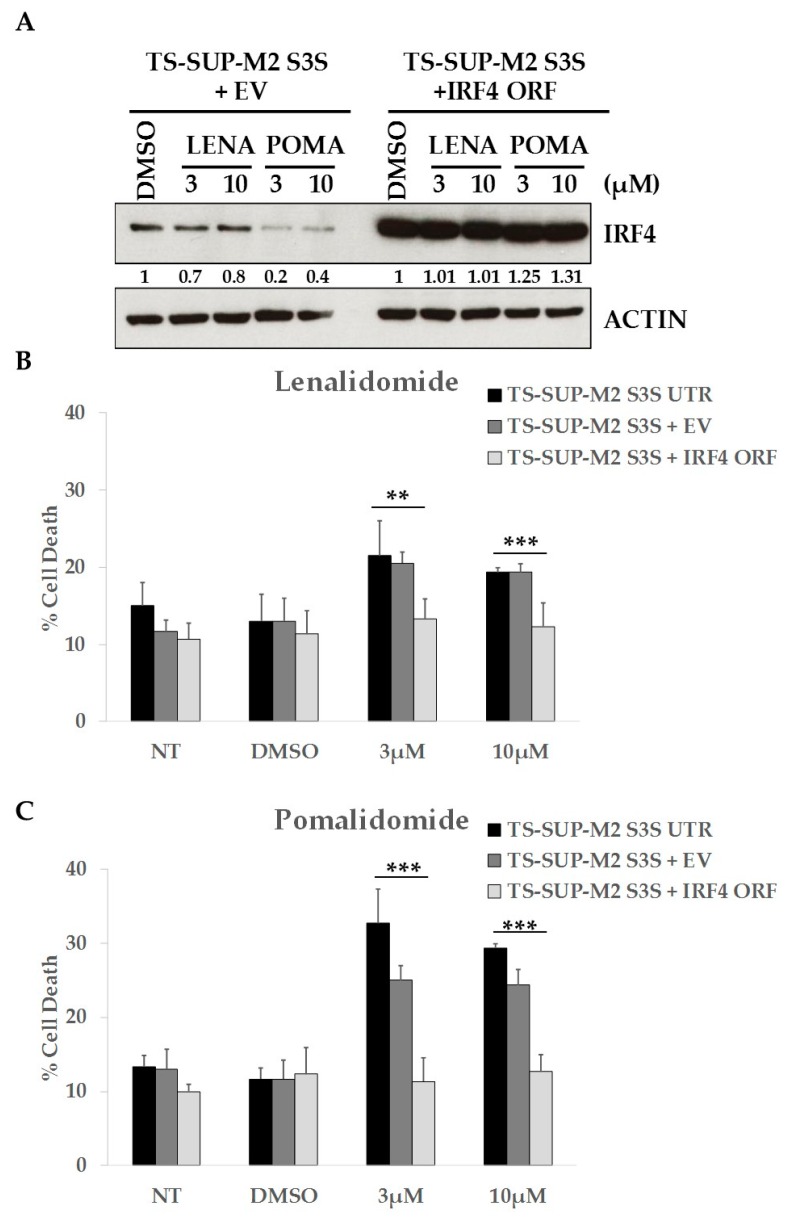
Immunomodulatory drugs downregulate IRF4 expression and increase cell death in TS-SUP-M2 cells. (**A**) TS-SUP-M2 S3S cells were transduced with human IRF4 ORF or with an empty vector (EV) and treated with the indicated concentrations of lenalidomide or pomalidomide. Western blot analysis revealed IRF4 downregulation after pomalidomide treatment both at 3 µM and 10 µM. Pellet for western blot were collected 4 days after treatment. Quantitative densiometric analysis were performed with ImageJ software. (**B**,**C**) Viability of TS-SUP-M2 S3S cells transduced with IRF4 ORF, empty vector (EV) or untransduced (UTR) as negative controls. Cells were treated with the indicated concentrations of lenalidomide, pomalidomide, diluent (DMSO), or left untreated (NT). Analysis of cell death revealed that ectopic expression of IRF4 completely rescued apoptosis induced by lenalidomide and pomalidomide. Apoptosis analysis was performed by TMRM staining-flow cytometry 6 days post treatments. Error bars represent the s.d. of triplicate measurements (** *p* < 0.01; *** *p* < 0.001).

It has been demonstrated that immunomodulatory drugs (IMiDs) such as lenalidomide and pomalidomide inhibit auto-ubiquitination of the E3 ubiquitin ligase cereblon (CRBN), leading to the degradation of Ikaros family targets and IRF4 [34,35,36]. We therefore wondered whether treatment with IMiDs could mimic IRF4 KD phenotype in ALCL cell lines. We observed that lenalidomide and pomalidomide treatments downregulated IRF4 expression and increased cell death in TS-SUP-M2 S3S cells. Conversely, overexpression of IRF4 completely rescued apoptosis induced by lenalidomide and pomalidomide (Figure 5), suggesting that IMiDs effects are mediated at least in part via IRF4 down-regulation. However, having extended these analyses to a larger number of ALCL cell lines, we observed that only TS-SUP-M2 S3S cells were sensitive to pomalidomide, as revealed by cell cycle, apoptosis, and cell metabolism markers (Figure S4). These data suggest that each ALCL may have different oncogenic addictions.

### 2.6. The Bromodomain and Extra-Terminal (BET)-Inhibitor JQ1 Sensitizes ALCL Cells to Pomalidomide Treatment

**Figure 6:**
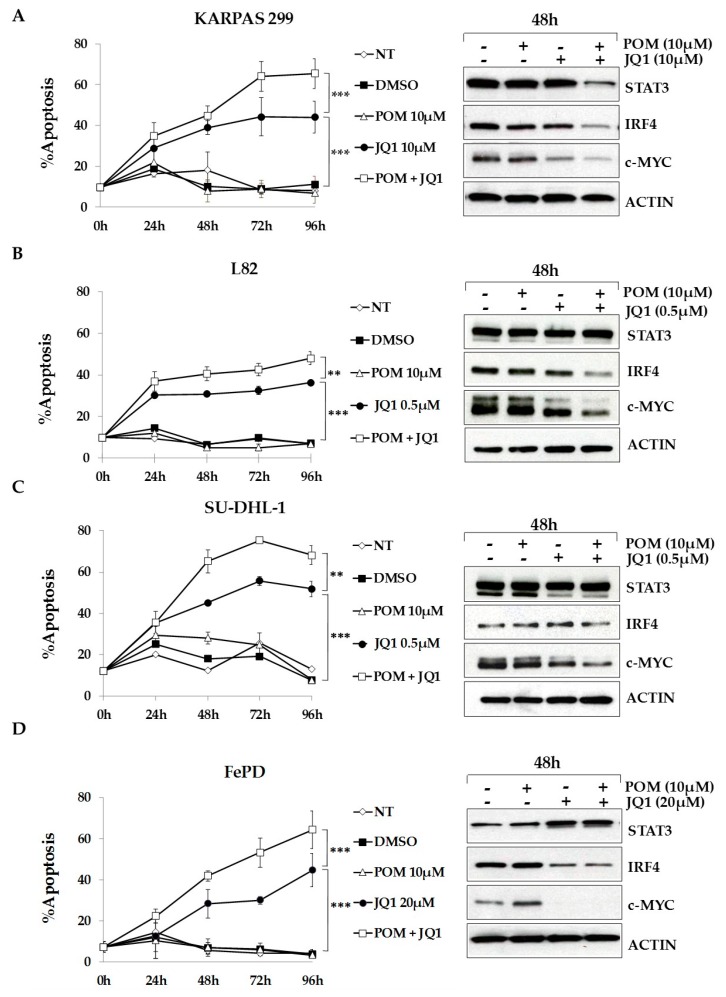
The bromodomain and extra-terminal (BET)-inhibitor JQ1 sensitizes ALCL cells to Pomalidomide treatment. Apoptosis and western blot analysis of KARPAS 299 (**A**), L82 (**B**), SU-DHL-1 (**C**) and FePd (**D**) cells treated with DMSO, pomalidomide, the BET inhibitor JQ1, or the combination of the two drugs. Cell death analysis revealed increased sensitivity to pomalidomide in combination with JQ1 (**left panels**) which correlates with a stronger down-regulation of IRF4 and c-MYC protein levels (**right panels**). Error bars represent the s.d. of triplicate measurements (** *p* < 0.01; *** *p* < 0.001). Pellet for western blot were collected 48 h after treatments.

Numerous studies have shown that c-MYC is essential for multiple myeloma cell survival and that IRF4 regulates its expression [37]. Moreover, it has been recently demonstrated that IRF4 and MYC signaling play an essential role ALCL cell lines survival [38] and that the treatment with BET family inhibitors may have a therapeutic efficacy in ALK positive ALCL [39]. Therefore, we tested whether the combination of pomalidomide with the BET family inhibitor JQ1, which inhibits MYC and IRF4 expression, could sensitize ALCL cells to IMiDs treatment. As shown in Figure 6, combination of the two drugs significantly increased cell death of Karpas-299, L82, SU-DHL-1 and FePd cells, as compared to single treatments. TS-SUP-M2 S3S, which are already sensitive to pomalidomide, did not show any significant change with the combination (Figure S5A). On the contrary, JB6 cells were resistant to both treatments (Figure S5B, left panel). Western blot analysis revealed that in this cell line the protein levels of IRF4 and c-MYC were not significantly downregulated by pomalidomide and JQ1 (Figure S5B, right panel). 

Paradoxically, we observed that JQ1 treatment resulted in the upregulation of IRF4 protein levels in JB6 cells, highlighting a possible feed-back loop between the two proteins, and potentially explaining the resistance of these cells to treatments. Taken together, these results show that IRF4 and MYC synergic inhibition can be exploited as therapeutic strategy in ALCLs.

## 3. Discussion

ALCL are a rare, heterogeneous group of non-Hodgkin lymphomas with an aggressive disease course and poor overall survival. Even though ALK positive and ALK negative ALCL show distinctive genomic alterations, the two entities are phenotypically similar and share significant biological and molecular key aspects. In particular, transcription factors activation, gene expression and epigenome profiles largely overlap in both ALCL entities [30,40,41,42,43,44], suggesting a common pathogenic mechanism. Among several oncogenic pathways, the constitutive activation of STAT3 is pathognomonic of ALK positive ALCL and it is detectable in ~50% ALK negative ALCL, as a consequence of JAK1/STAT3 mutations or aberrant cytokine receptor signalling [21]. STAT3 has been recognized a potentially promising target for therapy in ALCL and many other cancers. However, few STAT3 inhibitors achieved in vivo evaluation, and alternative targets in this pathway are currently under investigation [29]. 

Here, to dissect the STAT3 signaling network and to discover key players that may represent feasible targets for ALCL therapies, we performed a time course gene expression profiling experiment following conditional STAT3 knock-down. Transcriptional analysis identified 1730 genes specifically modulated by STAT3 silencing. The list included transcripts regulated by NPM-ALK dependent STAT3 signaling, as previously described [30,31]. Overall, more than 50% of the genes overlapped between the two studies. Notably, more than 60% of differentially expressed genes were up-regulated after STAT3 ablation, suggesting that STAT3 may also act as transcriptional repressor. Clustering analysis indicated that early-modulated genes were significantly enriched for conserved STAT3 binding sites in their regulatory regions, and therefore more likely to act as direct STAT3 targets. 

A functional screening by RNA interference targeting selected STAT3-regulated genes indicated that modulation of a single gene was usually not able to establish a remarkable phenotype in ALCL cells. A moderate growth disadvantage was induced either by BATF3 or ATF3 silencing (Table S3). These observations are in line with recent data which suggest that both ATF3 [9] and components of AP-1 transcription factor network act in concert, and a global AP-1 inhibition is required to cause death of ALCL cells [45]. Two genes (ITK and IRF4) showed a strong phenotype with multiple shRNAs. However, only IRF4 rescue assays restored cell viability and confirmed the specificity of shRNA experiments. Thus, we interpreted the toxicity caused by ITK silencing as a potential off-target effects related to shRNA sequences, and focused on IRF4. 

IRF4 is a member of the interferon regulatory factor family of transcription factors whose expression is restricted to the lymphoid and myeloid compartments and is not regulated by type I or type II interferons [46]. IRF4 was demonstrated to direct the development, maturation, and terminal differentiation of B cells and to play essential roles in orchestrating T cell fate decision [47,48]. Numerous evidences have implicated IRF4 as an oncogenic driver and potential target for inhibition by anti-cancer agents. The first association with cancer came from the identification of a chromosomal translocation that juxtaposes the immunoglobulin heavy-chain locus to *IRF4* in multiple myeloma patients [49]. Recurrent translocations involving *IRF4* locus were also identified in T-cell lymphomas such as PTCL-NOS and cutaneous ALCL [50]. Moreover, it was previously shown that the large majority of primary ALCL patient samples stained positive for IRF4 irrespective of ALK translocations, thus suggesting an important role for IRF4 in the pathogenesis of T-cell lymphomas [38]. Finally, IRF4 is required for the survival of myeloma cancer cells lacking *IRF4* translocations or overexpression, by a mechanism described as “non-oncogene addiction” [37]. 

Our experiments indicate that IRF4 knock down affects cell viability of ALK positive ALCL cells, thus suggesting that IRF4 could be involved in STAT3-mediated transformation and/or play a relevant role in the maintenance of the ALCL neoplastic phenotype. Analyses of IRF4 expression at transcriptional and protein level demonstrated that STAT3 knock-down is followed by IRF4 down-regulation. Computer analysis of the human IRF4 promoter revealed several putative STAT3 binding sites. These sites were found to be functional by STAT3 ChIP-seq experiments, supporting previous indications that IRF4 expression is under STAT3 control [48,51]. 

We further demonstrated that IRF4 partially contributes to the ALCL oncogenesis mediated via STAT3. In fact, cells transduced with human IRF4 exhibited lower apoptotic rate following STAT3 down-regulation. However, since the induction of apoptosis relies on a fine balance between pro- and anti-apoptotic proteins, the evaluation of IRF4 effect on the level of several survival regulating proteins should be more deeply investigated. 

Finally, to evaluate whether IRF4 signaling can be exploited therapeutically in the ALCL context, we tested the sensibility of several ALCL cells to IMiDs, such as lenalidomide and pomalidomide, that indirectly downregulate IRF4 [34,36,52]. Previous studies have shown an important role of IMiDs in numerous types of cancer, including multiple myeloma (MM), activated B-cell diffuse large B cell lymphoma (ABC-DLBCL), and acute myeloid leukemia (AML) [53,54,55,56]. IMiDs have numerous antitumoral effects, including immunomodulatory, anti-proliferative and pro-apoptotic effects [56]. We established the relationship between IMiDs and IRF4 in one ALK-positive ALCL cell line (TS-SUP-M2 S3S), and we found that lenalidomide and pomalidomide led to increased apoptosis. Moreover, IRF4 overexpression rescued pomalidomide toxic effects, highlighting the pivotal role of IRF4 in the survival of ALK-positive ALCL cells. However, further analysis revealed that only a minority of ALCL cell lines were sensitive to pomalidomide. In recent years, different studies have tried to explain IMiDs resistance. However, the molecular mechanisms are poorly understood and they may be related to the pharmacological properties of different drugs and the cell lineage. It has been previously reported that the deregulation of IMiDs’ targets, such as CRBN, or its substrates IRF4, IKZF1 and IKZF3 might affect the sensitivity to this class of drugs [34,52,53,57,58,59]. Moreover, C/EBPβ over-expression or β-catenin up-regulation have been described as additional mechanisms of IMiDs resistance [60,61]. In line with these observations, it is conceivable that also in ALCL IRF4-dependent and/or alternative mechanisms of IMiDs insensitivity might exist. 

It has been recently demonstrated that IRF4 and MYC signaling play an essential role ALCL cell lines survival [38,39]. Interestingly, the possibility of combinatorial block of MYC and IRF4 gene expression was greatly advanced by the demonstration that treatment of multiple myeloma tumor cells with the BET-bromodomain inhibitor JQ1 led to loss of BRD4 at super-enhancers, and consequent transcription elongation defects of genes with super-enhancers, including MYC and IFR4 [62]. Indeed, numerous studies have demonstrated, both in vitro and in vivo, the synergistic antitumor activity of IMiDs and BET-bromodomain inhibitors [63,64,65,66]. We observed that the combination of pomalidomide with the BET family antagonist JQ1 has additive effects in four of five pomalidomide-insensitive cell lines. Further analyses, as well as in vivo studies, are needed to confirm the efficacy of IMiDs and BET-bromodomain inhibitors combination therapy in ALCL and other tumors with de-regulated IRF4 [67]. Importantly, our data confirm the study by Weilemann et al., that demonstrated a key role of IRF4 in ALCL, irrespective of ALK status [38]. 

Here, we demonstrated the essential link between STAT3 and IRF4 expression, which could explain the sensitivity to IRF4 inhibition also in a fraction of ALK-negative ALCL [68]. This evidence is emphasized by the fact that STAT3 activation could be detected in almost 50% of ALK-negative ALCL [21]. On the other hand, previous works indicated that IRF4 is expressed in the majority (>90%) of primary ALCL cases [38,69]. These data are in agreement with the notion that IRF4 transcription could be activated by several factors such as NF-κB and STAT5 [38,70]. We proved that IRF4 mediates the oncogenic effects of STAT3 in ALCL, and speculated that its inhibition might represent an alternative avenue to interfere with STAT3 signaling. It is important to emphasize that IRF4 deficient mice have no obvious phenotypes outside of the lymphoid and myeloid lineages, in agreement with the restricted expression of IRF4 in these cell types. Therefore, potential therapies aimed at IRF4 inhibition are expected to be potentially manageable. In view of the above considerations, we ultimately envision that combination therapies using JAK/STAT3 inhibitors associated to BET-bromodomain inhibitors might offer a promising therapeutic strategy to overcome therapy resistance in ALCL patients.

## 4. Materials and Methods

### 4.1. Cell Lines and Culture Conditions

Human ALCL cells TS-SUP-M2 S3S, SU-DHL1, JB-6, KARPAS-299, FePd, and human T-ALL cells CCRF-CEM were cultured in RPMI 1640 (Sigma-Aldrich, St. Louis, MO, USA) medium supplemented with 10% fetal calf serum (Lonza, Rockland, ME, USA), 2 mM glutamine, 100 U/mL penicillin and 100 µg/mL streptomycin (Eurobio Biotechnology, Les Ulis, France). TS-SUP-M2 S3S (inducible cell line derived from TS-SUP-M2) were generated as described elsewhere [12,27,30]. Human embryonal kidney cells HEK-293T (ATCC, Manassas, VA, USA) were cultured in Dulbecco modified Eagle medium (DMEM) with identical supplements. Cell lines were incubated at 37 °C in humidified atmosphere, with 5% CO_2_.

### 4.2. shRNA Sequences, cDNA, and Plasmid Constructs

To stably knock down the expression of target genes we used specific shRNA cloned in the lentiviral vector pLKO-Puro. shRNAs (from A to E) (Table S4) were acquired from the Lentiviral Expression TRC Library (Sigma-Aldrich). Sense strand shRNA sequences for each gene are reported below. Replication-deficient lentiviral expression constructs for IRF4 were generated first by cloning the full-length cDNA of human IRF4 (acquired from Dharmacon, Lafayette, CO, USA) into pENTR1A no ccDB (Eric Campeau, http://ericcampeau.com/). Subsequently, lentiviral destination vector pLX303 h-IRF4 was generated recombining pENTRY vectors and pLX303 with LR reaction (Gateway System, Invitrogen, Carlsbad, CA, USA).

### 4.3. Virus Production and In Vitro Transduction

High titer lentiviral vector stocks were produced in HEK-293T cells by co-transfecting the expression vector (pLKO or pLX303) and the packaging vectors 8.74 and VSV-G/pMD2.G with the Effectene reagent (Qiagen, Hilden, Germany), according to the manufacturer’s instructions. Supernatants were harvested over 36 to 60 h, filtrated (0.22-µm pore), and used directly or after viral concentration by ultracentrifugation (50,000× *g* for 2 h). Virus titers were assessed by transducing TS-SUP-M2 cells with serial dilutions of viral stocks. Aliquots of virus were used to infect exponentially growing cells (1 × 10^5^/mL) in the presence of 8 µg/mL of polybrene. Fresh medium was supplemented 3–4 h after the infection. Stably transduced cells expressing shRNAs were selected by treatment with 2 µg/mL of puromycin (Sigma-Aldrich) for 24 h. The infectivity was determined (after 24 h) by FACS analysis of vital cells by TMRM staining-flow cytometry. For the reconstitution assay, exponentially growing TS-SUP-M2 cells were infected with 40 µL of lentiviral particles expressing human IRF4, or an empty vector (EV). Stably transduced cells were selected by treatment with 5 µg/mL Blasticidin (Sigma-Aldrich) for 48 h, expanded for 96 h, and plated for the infection with specific pLKO-Puro-shRNAs. 

### 4.4. Reverse Transcription-Quantitative Polymerase Chain Reaction (RT-qPCR)

Total RNA was extracted using the Trizol reagent (Invitrogen) or the RNeasy total RNA Isolation Kit (Qiagen), according to manufacturer’s instructions. RT-qPCR was performed with a Thermal iCycler (Bio-Rad Laboratories, Hercules, CA, USA) using the iQ SYBR Green Supermix (Bio-Rad Laboratories) according to the manufacturer’s instructions. The PCR cycling conditions were as follows: 95 °C for 5 min followed by 40 cycles at 94 °C for 10 s and 60 °C for 30 s. The oligonucleotide primer pairs used for RT-qPCR, reported in Table S5, were designed to obtain amplicons of 80–150 bp. To confirm the amplification specificity, the PCR products were subjected to the analysis of melting curve, linearity and slope of standard curve. All PCR assays were performed in triplicate. The results were analyzed using the comparative ΔCt method as described by Schmittgen and Livak [71].

### 4.5. Immunoblotting

Whole cell extracts were prepared by resuspending the cell pellets in lysis buffer containing 20 mM Tris-HCl (pH 7.4), 150 mM NaCl, 5 mM EDTA, 0.1% Triton X-100, 1 mM phenyl-methyl-sulfonyl fluoride (PMSF), 10 mM NaF, 1 mM Na_3_VO_4_, and protease inhibitors (Roche, Mannheim, Germany) and incubated at 4 °C for 30 min. Cell lysates were collected by centrifugation at 15,000× *g*. Supernatants were analyzed for protein concentration with a DC protein assay kit (Bio-Rad) and stored at −80 °C. Twenty micrograms of proteins were separated by sodium dodecyl sulfate-polyacrylamide gel electrophoresis (SDS-PAGE) and transferred onto nitrocellulose membranes. The filters were first blocked for 1 hour at room temperature with 5% low-fat milk in phosphate-buffered saline (PBS) solution with 0.1% Tween 20, and then incubated with the primary antibodies for 1 h at room temperature. After 3 washes, filters were incubated with horseradish peroxidase-conjugated goat anti-mouse or anti-rabbit antibodies (1:10,000; Amersham, Arlington Heights, IL, USA) for 1 h at room temperature. Immune complexes were detected with sheep-anti mouse or anti-rabbit Ig antibodies conjugated to horseradish peroxidase (Amersham) and visualized by enhanced chemiluminescence reagent (Amersham) according to the manufacturer’s protocol. The following antibodies were used: mouse anti-β-tubulin (#T4026); rabbit anti-actin (#A5060) (Sigma-Aldrich); rabbit anti-c-MYC (Cell Signaling, Danvers, MA, USA); mouse anti-STAT3 (Zymed, South San Francisco, CA, USA), mouse anti-IRF4 (Agilent, Santa Clara, CA, USA), rabbit anti-PARP H-250 (Santa Cruz Biotechnology, Dallas, TX, USA), rabbit anti-cyclin A (H-432) (Santa Cruz Biotechnology), rabbit anti-cyclin B (H-433) (Santa Cruz Biotechnology), mouse anti-p27[kip-1] (BD Biosciences, San Jose, CA, USA).

### 4.6. Flow Cytometry Analysis of Apoptosis and Cell Cycle

Apoptosis was measured by flow cytometry after staining with the mitochondrion-permeable, voltage sensitive dye Tetrametylrodamine methyl ester (TMRM, Molecular Probes, Eugene, OR, USA) [68]. 5 × 10^5^ cells were washed once in phosphate-buffered saline (PBS), incubated for 15 min at 37 °C in HEPES buffer solution (10 mM HEPES pH 7.4, 140 mM NaCl, 2.5 mM CaCl) with 200 nM TMRM. Cells were analyzed by FACSCalibur, using CellQuest software (BD Pharmingen Biosciences, San Jose, CA, USA). For cell cycle analysis and DNA content determination, cells were fixed for 1 h in 70% ethanol at 4 °C. After washing with PBS, cells were treated with RNase (0.25 mg/mL), stained with propidium iodide (50 µg/mL) for 30 min. Then cells were analyzed by FACSCalibur. The subG_1_/G_0_-phase fraction was calculated using CellQuest program (BD Pharmingen Biosciences). 

### 4.7. ATPlite Assay

ATPlite assay was performed using CellTiter-Glo^®^ Luminescent Cell Viability Assay (Promega, Madison, WI, USA) to measure cell viability/proliferation. For each experiment cells were plated at a density of 1 × 10^5^/mL and cell proliferation was measured at day 0, day 3 and 5. 50 µL of cells were mixed with an equal volume of CellTiter-Glo^®^ Reagent solution per well in 96-well black plate, in triplicate. Plate was shaken in the dark for 2 min and then incubated at room temperature for 10 min. Luminescence was measured using a TopCount NXT Microplate Scintillation and Luminescence Counter (Packard BioScence Company, Meriden, CT, USA).

### 4.8. Lenalidomide, Pomalidomide and JQ1 Treatments

Lenalidomide, pomalidomide and JQ1 were obtained from Selleck Chemicals (Houston, TX, USA). Compounds were dissolved in 100% dimethyl sulfoxide (DMSO) before further dilution in cell culture media. Final DMSO concentrations were manteined at 0.1% for all samples, including controls. Exponentially growing cells were plated at a density of 1 × 10^5^/mL and treated with increasing doses of lenalidomide, pomalidomide or JQ1 (with pomalidomide treatment refilled every 48 h). Apoptosis and cell proliferation was measured by flow cytometry after staining with the mitochondrion-permeable voltage sensitive dye tetrametylrodamine methyl ester (TMRM) and ATPlite assay, respectively. At the indicated time points pellet was collected and whole-cell extracts were subjected to western blot analysis.

### 4.9. Gene Expression Profiling

TS-SUP-M2 S3S were treated with doxycycline (1 mg/mL) to induce short shRNA expression, and monitored for green fluorescent protein (GFP) expression by FACS analysis. STAT3 was monitored by western blotting. Biological triplicates were used for each experimental condition. Total RNA was isolated using the Trizol reagent (Invitrogen) and purified using the RNeasy total RNA Isolation Kit (Qiagen). RNA integrity was evaluated by an Agilent 2100 Bioanalyzer (Agilent Technologies, Palo Alto, CA, USA). cDNA and biotinylated cRNAs were generated by Illumina Total Prep RNA Amplification Kit (Ambion, Austin, TX, USA), in accordance to manufacturer’s indications. cRNAs quality and quantification was assessed by Bioanalyzer. Hybridization was carried out on HumanHT-12 v4.0 Expression BeadChip (Illumina Inc.). Array washing, staining and scanning were performed using standard Illumina protocols. Detection data were processed with the BeadStudio software (Illumina Inc.) using the following thresholds for significant detection: Differential Score > 30 (equivalent to *p* < 0.001), Detection > 0.99, Fold Change > 2.

### 4.10. Chromatin Immunoprecipitation-Sequencing (ChIP-seq)

A total of 4 × 10^6^ cells were fixed with 1% formaldehyde, lysed, and sonicated (Branson Sonicator; Branson Ultrasonics, Danbury, CT, USA) leading to a DNA average size of 200 bp. 5 µg of antibodies anti-STAT3 #4904 (Cell Signaling), H3K4me3 (ab8580, Abcam, Cambridge, UK) H3K27Ac (ab177178, Abcam), H3K27me3 (ab6002, Abcam), or control IgG #2729 (Cell Signaling) were added to the precleared sample and incubated overnight at 4  °C. The complexes were purified using CHIP-grade protein-G magnetic beads #9006 (Cell Signaling), followed by elution from the beads and reverse cross-linking. DNA was purified using PCR purification columns (Qiagen) and target control genes (*TNFRSF8* and *GAPDH* were amplified by real-time quantitative PCR using SYBR Green (Bio-Rad, #1725272). The oligonucleotide primer pairs are reported in Table S5. Raw ChIP-Seq samples were aligned using bwa aligner. Peak calling was performed using Model-based Analysis of ChIP-Seq (MACS Version 2.1.1, [72]), an open source computational algorithm for identifying genome-wide protein-DNA interaction from ChIP-Seq data (https://github.com/taoliu/MACS). Only peaks with FDR < 0.05 were called as significantly enriched. Signal tracks were created using MACS2 bdgcmp command. Peak enrichment relative to IgG was calculated in bedgraph format and then converted to bigwig using UCSC toolkit. 

### 4.11. Statistics

Statistical analysis was performed with a paired, 2-tailed Student *t* test. *p* < 0.05 were considered statistically significant. Data presented with column graphs and error bars represent average ± SD.

## 5. Conclusions

Overall, these data indicate that IRF4 sustains the oncogenic properties of STAT3 in T-cell lymphomas, and that its inhibition represents an alternative avenue to interfere with STAT3 signaling and offers promising therapeutic opportunities to treat and prevent drug resistance in ALCL patients. 

## Supplementary Materials

The following are available online at http://www.mdpi.com/2072-6694/10/1/21/s1. Figure S1: Kinetics of STAT3-regulated genes in the ALK-positive ALCL cell line TS-SUP-M2. Figure S2: Enrichment of putative STAT3 binding sites in cluster 7 (early down-regulated genes). Figure S3: STAT3 regulates IRF4 expression in ALCL cells. Figure S4: Effects of Pomalidomide treatment in ALCL cell lines. Figure S5: Effects of Pomalidomide combination with the BET family inhibitor JQ1 in SUP-M2 S3S and JB6 cell lines. Table S1: List of Cluster 7 genes. Table S2: Cluster 7 genes selected for functional screening with shRNA. Table S3: Schematic result of the functional shRNA screening on cluster 7 genes. Table S4: List of shRNA sequences utilized in the screening. Table S5: Primer sequences used in the present study.
