# Supplementary material for: IRF4 Mediates the Oncogenic Effects of STAT3 in Anaplastic Large Cell Lymphomas
Source: Cancers (Basel). 2018 Jan 18;10(1):21. doi: 10.3390/cancers10010021 (PMC5789371; doi:10.3390/cancers10010021)
Supplement: Supplementary file 1 [file cancers-10-00021-s001.pdf]

# Supplementary Materials: IRF4 Mediates the Oncogenic Effects of STAT3 in Anaplastic Large Cell Lymphomas

**Cecilia Bandini, Aldi Pupuleku, Elisa Spaccarotella, Elisa Pellegrino, Rui Wang, Nicoletta Vitale, Carlotta Duval, Daniela Cantarella, Andrea Rinaldi, Paolo Provero, Ferdinando Di Cunto, Enzo Medico, Francesco Bertoni, Giorgio Inghirami, Roberto Piva**

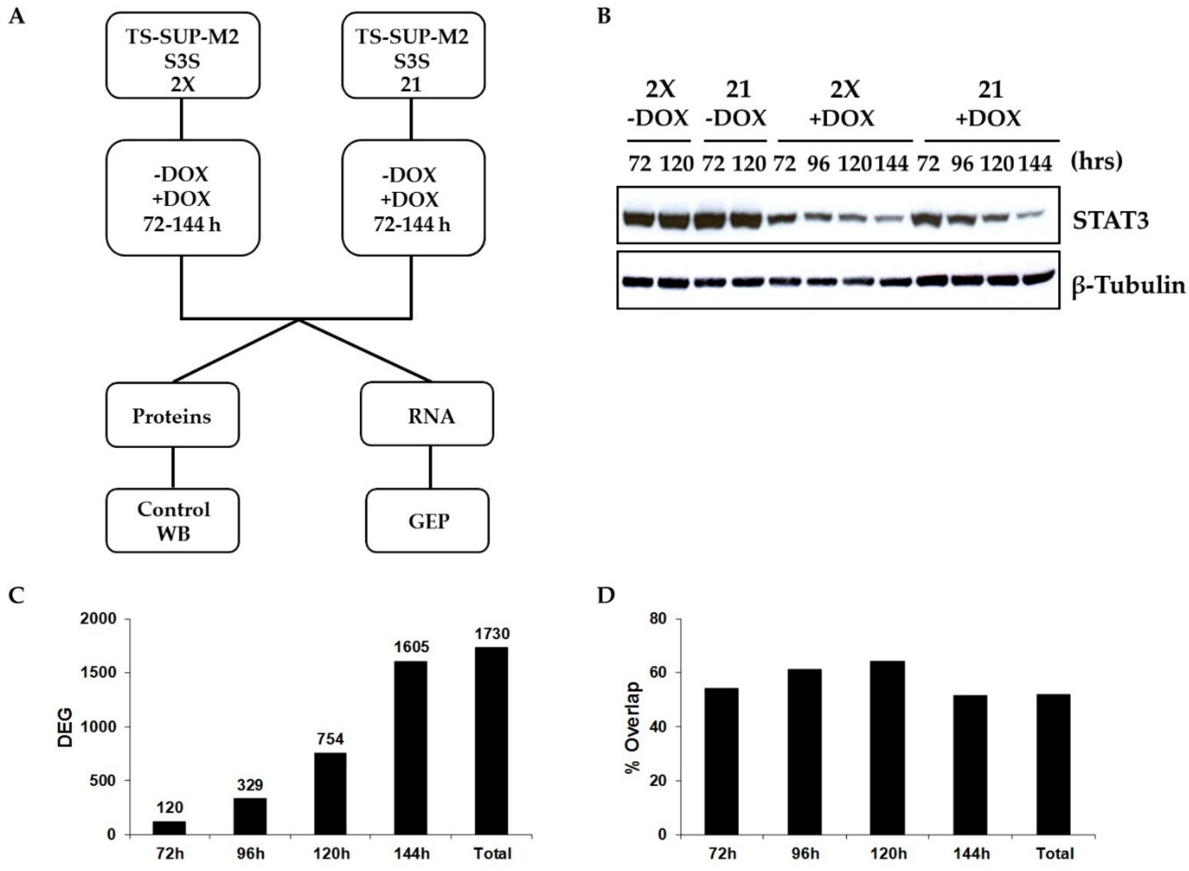


**Figure S1.** Kinetics of STAT3-regulated genes in the ALK-positive ALCL cell line TS-SUP-M2. **(A)** Experimental design of the gene expression profile (GEP) experiment. Two clones (2X and 21) of the ALKpositive ALCL cell line (TS-SUP-M2 S3S) expressing a specific doxycycline-inducible STAT3 short hairpin RNA (shRNA) were used [27]. Biological triplicates of TS-SUP-M2 S3S cells were cultured in the presence (+DOX) or absence (−DOX) of doxycycline (1 μg/mL) for 0, 72, 96, 120, 144 h, and harvested for RNA and protein extraction. RNA was labeled by Illumina Total Prep RNA Amplification Kit, and hybridized on Illumina HumanHT-12 BeadChip Array. **(B)** STAT3 silencing was confirmed by western blotting at the indicated time points. **(C)** Differential Expressed Genes (DEG) for each time point. In total 1730 genes were modulated after STAT3 KD. Differential score: $p < 0.001$ and FC > 2. **(D)** Percentage of overlapping DEG with a previous STAT3 knock down GEP experiment performed with 3 different shRNAs [30].

**A**

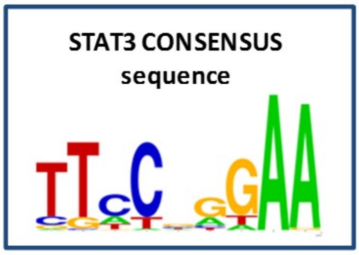


**B**

| | stat3.1 | stat3.2 | stat3.4 | stat3.6 | stat3.8 |
|---|---|---|---|---|---|
| cluster_01 | n=79 (68.0) P=0.057 | n=37 (38.9) P=0.66 | n=12 (7.0) P=0.049 | n=3 (1.7) P=0.25 | n=0 (0.3) P=1 |
| cluster_02 | n=57 (51.6) P=0.2 | n=31 (29.5) P=0.41 | n=4 (5.3) P=0.78 | n=0 (1.3) P=1 | n=0 (0.2) P=1 |
| cluster_03 | n=57 (50.9) P=0.16 | n=29 (29.1) P=0.54 | n=6 (5.3) P=0.43 | n=1 (1.3) P=0.73 | n=0 (0.2) P=1 |
| cluster_04 | n=55 (52.3) P=0.35 | n=36 (29.9) P=0.13 | n=7 (5.4) P=0.3 | n=2 (1.3) P=0.39 | n=0 (0.2) P=1 |
| cluster_05 | n=59 (54.1) P=0.22 | n=32 (30.9) P=0.44 | n=5 (5.6) P=0.66 | n=2 (1.4) P=0.4 | n=0 (0.2) P=1 |
| cluster_06 | n=8 (10.5) P=0.87 | n=3 (6.0) P=0.96 | n=1 (1.1) P=0.67 | n=1 (0.3) P=0.24 | n=0 (0.0) P=1 |
| cluster_07 | n=82 (61.4) P=0.00082 | n=54 (35.1) P=0.00043 | n=8 (6.3) P=0.3 | n=3 (1.6) P=0.21 | n=1 (0.3) P=0.23 |
| cluster_08 | n=32 (34.5) P=0.74 | n=10 (19.7) P=1 | n=1 (3.6) P=0.97 | n=0 (0.9) P=1 | n=0 (0.1) P=1 |
| cluster_09 | n=23 (26.5) P=0.83 | n=14 (15.1) P=0.67 | n=3 (2.7) P=0.52 | n=1 (0.7) P=0.5 | n=0 (0.1) P=1 |
| cluster_10 | n=47 (46.0) P=0.46 | n=24 (26.3) P=0.73 | n=3 (4.8) P=0.86 | n=1 (1.2) P=0.7 | n=1 (0.2) P=0.18 |
| cluster_11 | n=59 (48.8) P=0.044 | n=33 (27.9) P=0.16 | n=6 (5.0) P=0.39 | n=2 (1.3) P=0.36 | n=1 (0.2) P=0.18 |
| cluster_12 | n=27 (22.3) P=0.14 | n=16 (12.8) P=0.19 | n=2 (2.3) P=0.68 | n=2 (0.6) P=0.11 | n=0 (0.1) P=1 |

**Figure S2.** Enrichment of putative STAT3 binding sites in cluster 7 (early down-regulated genes). (**A**) The position weight matrix (PWM) STAT3 sequence logo obtained as described by Vallania et al, 2009 [32]. (**B**) The number of genes carrying putative STAT3 binding sites is represented for each cluster of genes regulated by STAT3 inducible knockdown in the cell line TS-SUP-M2 S3S (P = *p* value). Each human gene is associated with a score which is the sum of the conservation scores of the STAT3 sites found in its promoter region (−2000 +500 from TSS).The enrichment is evaluated with respect to all the genes in the chip that were detected above background in at least one sample. Cluster 7 showed a strong overrepresentation of putative STAT3 targets. This cluster includes 82 genes bearing one STAT3 binding site (stat3.1; *p* value 0.00082) and 54 genes with two binding sites (stat3.2; *p* value 0.00043).

**A**

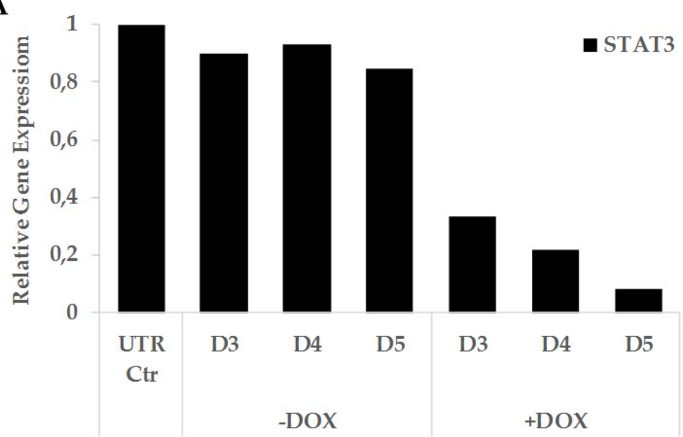


**B**

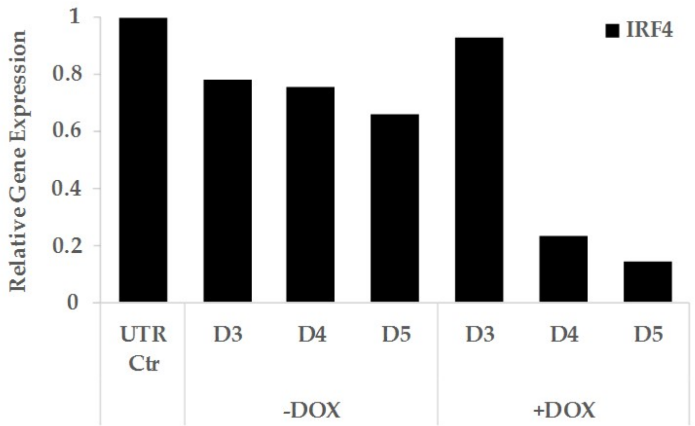


**C**

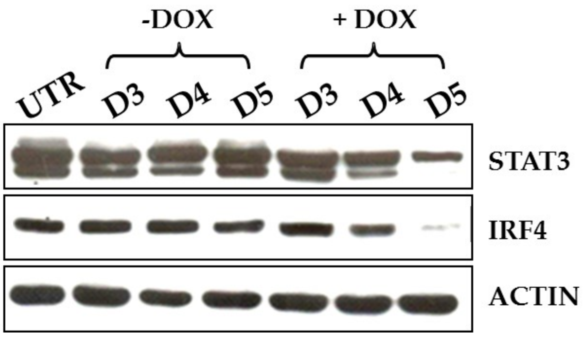


**Figure S3.** STAT3 regulates IRF4 expression in ALCL cells. TS-SUP-M2 S3S cells were grown in the absence (-DOX) or presence (+DOX) of doxycycline (1μg/mL) to induce STAT3 depletion. Pellet for RT-qPCR and western blot analyses were collected at 72 (D3), 96 (D4) and 120 (D5) h. (**A**–**B**) RT-qPCR analysis shows progressive decrease of STAT3 and IRF4 mRNA levels after 72 and 96 h of doxycycline treatment, respectively. (**B**) RT-qPCR analysis shows progressive decrease of IRF4 mRNA levels after 96 h of doxycycline treatment. Pellet were collected at 72 (D3), 96 (D4) and 120 (D5) h. TS-SUP-M2 S3S were grown in the presence (+DOX) or absence (−DOX) of doxycycline (1 μg/mL) for 120 h. TS-SUP-M2 S3S cells showed remarkable STAT3 and IRF4 downregulation after 5 days treatment with doxycycline. (**A**) STAT3 silencing after 5 days treatment with doxycycline detected by RT-qPCR. (**B**) IRF4 mRNA levels significantly decreased following STAT3 downregulation. (**C**) Western blot analysis showing progressive decrease of STAT3 and IRF4 protein levels after doxycycline treatment.

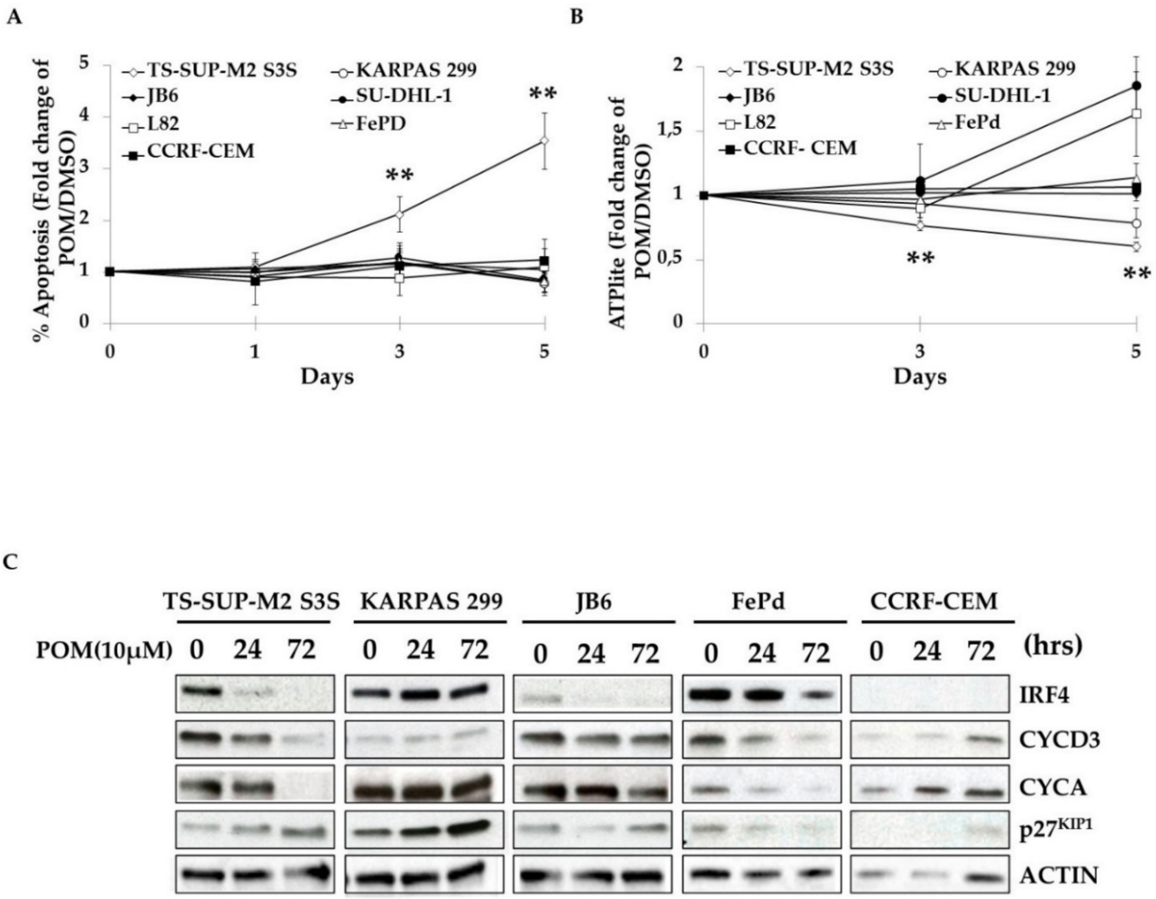


**Figure S4.** Effects of Pomalidomide treatment in ALCL cell lines. (**A**) Cell viability and (**B**) proliferation were measured in different ALCL cell lines at the indicated time points after DMSO or Pomalidomide (10μM) treatment. T-ALL CCRF-CEM cells were used as negative control. Data are presented as mean ± SD ($n$ = 5). Analysis of cell death and cell proliferation revealed that only TS-SUP-M2 S3S cells were significantly sensitive to Pomalidomide treatment (**$p$ < 0.01;***$p$ < 0.001). (**C**) Western blot analysis of cells after Pomalidomide treatment at the indicated time points.

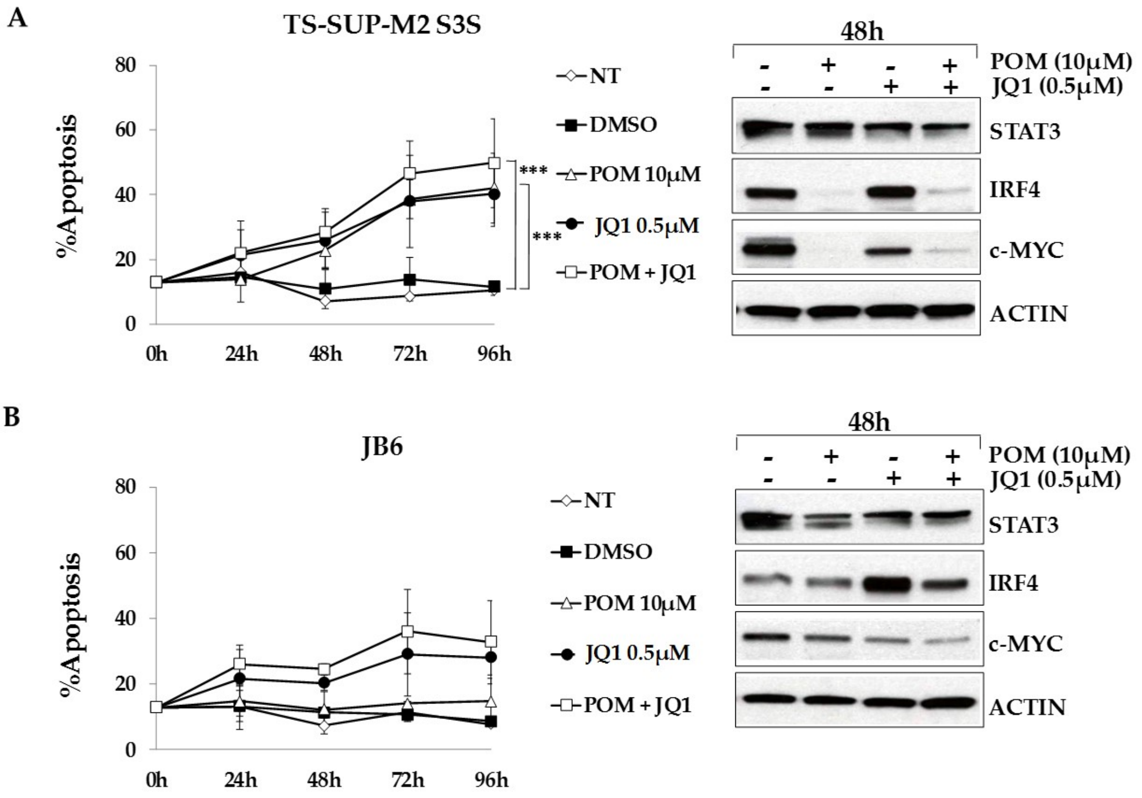


**Figure S5.** Effects of Pomalidomide combination with the BET family inhibitor JQ1 in TS-SUP-M2 S3S and JB6 cell lines. Apoptosis and western blot analysis of TS-SUP-M2 S3 (**A**) and JB6 (**B**) cells treated with DMSO, Pomalidomide, the BET inhibitor JQ1, or the combination of the two drugs. Error bars represent the s.d. of triplicate measurements (***p* < 0.01;****p* < 0.001). Pellet for western blot were collected 48 hours after treatments.

**Table S1.** List of Cluster 7 genes. Cluster 7 includes transcripts early down-regulated following inducible STAT3 KD in TS-SUP-M2cells

| TargetID | ProbeID | SEARCH_KEY | CLUSTER ID | −DOX AVG_Sig | +DOX 72 h Log2R | +DOX 96 h Log2R | +DOX 120 h Log2R | +DOX 144 h Log2R | Oldexp |
|---|---|---|---|---|---|---|---|---|---|
| HS.527588 | 870047 | ILMN_100916 | 7 | 3049 | −2.78 | −3.68 | −3.95 | −4.05 | |
| C13ORF16 | 3390041 | ILMN_19109 | 7 | 8732 | −2.54 | −3.52 | −4.19 | −5.08 | |
| FGB | 7210240 | ILMN_13882 | 7 | 2650 | −2.40 | −3.24 | −3.46 | −3.84 | −1.92 |
| ZNF395 | 1980403 | ILMN_17231 | 7 | 4559 | −2.28 | −1.62 | −2.03 | −2.81 | −2.28 |
| TNFRSF9 | 5670440 | ILMN_13404 | 7 | 870 | −2.13 | −1.97 | −2.18 | −2.06 | −0.74 |
| LEF1 | 2810601 | ILMN_30265 | 7 | 7327 | −2.09 | −2.90 | −3.42 | −3.80 | −2.54 |
| SLC2A5 | 7560541 | ILMN_14785 | 7 | 6049 | −2.08 | −1.91 | −1.75 | −2.25 | |
| ANKRD37 | 240682 | ILMN_2423 | 7 | 2756 | −2.07 | −1.36 | −1.32 | −2.45 | −1.65 |
| UHRF2 | 2970612 | ILMN_138390 | 7 | 2497 | −2.02 | −2.01 | −1.64 | −1.99 | −1.30 |
| LEF1 | 4570255 | ILMN_30265 | 7 | 6489 | −1.99 | −2.97 | −3.39 | −3.82 | −2.54 |
| ICOS | 2070037 | ILMN_9996 | 7 | 13,683 | −1.99 | −3.00 | −2.98 | −3.38 | −2.41 |
| PSCD4 | 6510524 | ILMN_16536 | 7 | 8740 | −1.98 | −2.30 | −2.51 | −2.20 | −1.49 |
| LOC196549 | 2710717 | ILMN_17912 | 7 | 1588 | −1.97 | −1.57 | −1.83 | −2.44 | −1.60 |
| FCRLB | 4730204 | ILMN_24322 | 7 | 4927 | −1.93 | −1.88 | −1.47 | −1.74 | −1.06 |
| LOC145837 | 4050674 | ILMN_30561 | 7 | 1311 | −1.90 | −2.33 | −2.48 | −2.49 | |
| STARD13 | 3440184 | ILMN_4567 | 7 | 2932 | −1.89 | −1.93 | −2.38 | −3.18 | −1.13 |
| IL6R | 5870685 | ILMN_6641 | 7 | 940 | −1.89 | −2.17 | −2.21 | −2.58 | −1.36 |
| SGK | 4390450 | ILMN_2451 | 7 | 5883 | −1.85 | −2.33 | −2.56 | −2.94 | −2.20 |
| STAT3 | 5090619 | ILMN_29673 | 7 | 15,774 | −1.80 | −1.96 | −2.32 | −2.75 | −1.94 |
| SPTLC3 | 6590402 | ILMN_90698 | 7 | 1223 | −1.76 | −1.77 | −1.64 | −2.17 | |
| LOC158160 | 2030672 | ILMN_21155 | 7 | 22,170 | −1.76 | −2.94 | −3.47 | −3.65 | −0.81 |
| SPTLC3 | 3780743 | ILMN_7329 | 7 | 1177 | −1.75 | −1.84 | −1.85 | −2.16 | |
| UPK1B | 4490068 | ILMN_11904 | 7 | 7281 | −1.72 | −2.23 | −3.19 | −3.63 | −2.24 |
| HS.560343 | 6400564 | ILMN_114182 | 7 | 2546 | −1.71 | −1.90 | −2.12 | −1.86 | |

| | | | | | | | | | |
|---|---|---|---|---|---|---|---|---|---|
| STARD13 | 3190411 | ILMN_4466 | 7 | 1160 | −1.69 | −1.63 | −1.91 | −2.36 | |
| STAT3 | 2100484 | ILMN_170837 | 7 | 4524 | −1.68 | −1.77 | −2.20 | −2.68 | |
| ALB | 650431 | ILMN_28973 | 7 | 857 | −1.67 | −2.09 | −2.16 | −2.37 | |
| HS.93739 | 4540446 | ILMN_74213 | 7 | 670 | −1.65 | −1.58 | −1.42 | −1.69 | |
| STAT3 | 4250538 | ILMN_29673 | 7 | 4178 | −1.61 | −1.95 | −2.25 | −2.64 | −1.94 |
| TMPRSS11D | 2320168 | ILMN_28873 | 7 | 2562 | −1.60 | −2.35 | −2.57 | −3.48 | −1.94 |
| MMP20 | 2480390 | ILMN_1399 | 7 | 747 | −1.56 | −1.69 | −1.81 | −2.17 | −1.50 |
| HS.37648 | 4860280 | ILMN_72502 | 7 | 1541 | −1.53 | −1.59 | −1.69 | −1.71 | |
| IL10 | 6180093 | ILMN_9173 | 7 | 878 | −1.46 | −1.63 | −2.26 | −2.25 | |
| HSD17B7P2 | 7610142 | ILMN_1267 | 7 | 4835 | −1.44 | −2.86 | −3.41 | −3.28 | −2.21 |
| HS.448059 | 2510671 | ILMN_93404 | 7 | 2244 | −1.42 | −1.56 | −1.48 | −1.68 | |
| SLCO2B1 | 6580441 | ILMN_1897 | 7 | 820 | −1.42 | −1.81 | −1.72 | −1.82 | −1.08 |
| MEIS2 | 20358 | ILMN_3095 | 7 | 1581 | −1.41 | −1.36 | −1.07 | −1.11 | −1.36 |
| AXUD1 | 7560041 | ILMN_6524 | 7 | 9353 | −1.40 | −1.07 | −0.95 | −1.08 | −0.79 |
| IGSF11 | 5270102 | ILMN_551 | 7 | 1617 | −1.40 | −1.45 | −1.85 | −2.38 | |
| ABCA13 | 1780537 | ILMN_19037 | 7 | 9378 | −1.35 | −0.64 | −0.09 | −0.07 | −0.68 |
| TNFRSF21 | 3780092 | ILMN_9651 | 7 | 10,608 | −1.35 | −1.15 | −1.53 | −1.74 | −1.50 |
| HS.529631 | 7200286 | ILMN_101314 | 7 | 1780 | −1.33 | −1.83 | −1.59 | −1.94 | |
| FAM46C | 6860347 | ILMN_7706 | 7 | 8119 | −1.32 | −1.17 | −0.69 | −1.36 | −0.84 |
| CXCR5 | 3890400 | ILMN_19896 | 7 | 577 | −1.32 | −1.37 | −1.35 | −1.67 | |
| CMAH | 5550066 | ILMN_13868 | 7 | 5192 | −1.31 | −1.21 | −1.33 | −1.92 | |
| NSL1 | 4640370 | ILMN_164300 | 7 | 701 | −1.31 | −1.65 | −1.94 | −1.91 | |
| CD59 | 1410201 | ILMN_1905 | 7 | 953 | −1.30 | −1.51 | −1.14 | −0.88 | |
| FBXO32 | 1990079 | ILMN_3809 | 7 | 5857 | −1.30 | −1.06 | −0.48 | −0.70 | −0.72 |
| LOC642299 | 6020561 | ILMN_31918 | 7 | 1926 | −1.29 | −1.51 | −1.47 | −1.72 | |
| IQCG | 70551 | ILMN_26972 | 7 | 7879 | −1.28 | −1.91 | −2.07 | −1.88 | −2.07 |
| RORA | 1110180 | ILMN_13528 | 7 | 2362 | −1.28 | −1.77 | −1.95 | −2.10 | −1.55 |
| HS.36053 | 2940228 | ILMN_72440 | 7 | 2285 | −1.24 | −1.86 | −1.84 | −2.24 | |
| MYBPC2 | 5270100 | ILMN_16419 | 7 | 10,920 | −1.21 | −1.91 | −1.89 | −2.22 | −1.55 |
| CD5 | 5050347 | ILMN_29547 | 7 | 1644 | −1.20 | −1.68 | −2.15 | −2.22 | −1.25 |
| SOX2 | 5130156 | ILMN_171554 | 7 | 1801 | −1.20 | −1.78 | −1.66 | −1.87 | |
| NFIL3 | 5420564 | ILMN_14880 | 7 | 3162 | −1.19 | −0.71 | −0.69 | −0.81 | −1.19 |
| PHF21A | 6580164 | ILMN_28019 | 7 | 8593 | −1.18 | −0.71 | −0.22 | −0.29 | |
| HS.190748 | 1260356 | ILMN_81073 | 7 | 612 | −1.17 | −1.29 | −1.37 | −1.23 | |
| LOC651621 | 630095 | ILMN_45641 | 7 | 2196 | −1.17 | −2.06 | −2.26 | −2.29 | |
| TGM2 | 2940446 | ILMN_8134 | 7 | 18,359 | −1.16 | −0.77 | −0.69 | −0.68 | |
| CERK | 3060692 | ILMN_2275 | 7 | 3138 | −1.16 | −0.79 | −0.63 | −0.85 | |
| GZMB | 1850523 | ILMN_23555 | 7 | 40,169 | −1.15 | −2.45 | −3.11 | −3.45 | −1.34 |
| VCL | 70592 | ILMN_26712 | 7 | 16,372 | −1.13 | −1.60 | −1.21 | −1.21 | |
| CMAH | 4250634 | ILMN_13868 | 7 | 3642 | −1.13 | −1.36 | −1.24 | −1.83 | |
| C9ORF38 | 50209 | ILMN_36611 | 7 | 978 | −1.12 | −1.49 | −1.48 | −1.99 | |
| SEMA4B | 5080280 | ILMN_25026 | 7 | 1832 | −1.12 | −0.47 | −0.75 | −0.70 | |
| RGS16 | 1030102 | ILMN_16445 | 7 | 5498 | −1.11 | −1.43 | −2.25 | −2.08 | −1.65 |
| TNFRSF10D | 830113 | ILMN_17600 | 7 | 1101 | −1.10 | −1.15 | −0.71 | −0.77 | |
| STAMBPL1 | 7150059 | ILMN_1387 | 7 | 6426 | −1.10 | −0.23 | 0.08 | −0.65 | |
| EDG1 | 160754 | ILMN_138993 | 7 | 581 | −1.10 | −1.35 | −1.43 | −1.62 | −1.78 |
| CCDC46 | 6400161 | ILMN_173004 | 7 | 2803 | −1.10 | −1.46 | −0.98 | −1.02 | |
| RORA | 3940703 | ILMN_13528 | 7 | 808 | −1.10 | −1.59 | −1.57 | −1.72 | −1.55 |
| ERC2 | 7150050 | ILMN_17447 | 7 | 605 | −1.10 | −1.24 | −0.65 | −0.39 | −0.60 |
| LOC440895 | 5310575 | ILMN_44926 | 7 | 799 | −1.10 | −0.86 | −0.82 | −1.50 | |
| ALB | 5910010 | ILMN_28973 | 7 | 581 | −1.10 | −1.58 | −1.68 | −1.44 | |
| FLJ22447 | 1240653 | ILMN_37423 | 7 | 1117 | −1.09 | −1.34 | −1.43 | −1.75 | |
| CDK5RAP2 | 7570348 | ILMN_9876 | 7 | 784 | −1.09 | −1.62 | −1.69 | −1.79 | −0.98 |
| TNFRSF8 | 4850253 | ILMN_21182 | 7 | 6392 | −1.08 | −0.95 | −1.77 | −2.34 | −1.36 |
| C5ORF13 | 940471 | ILMN_2442 | 7 | 13,584 | −1.08 | −1.11 | −1.15 | −1.18 | −1.33 |
| BIRC3 | 5080021 | ILMN_3897 | 7 | 2415 | −1.07 | −0.81 | −0.24 | 0.07 | |
| CFH | 380424 | ILMN_12111 | 7 | 3249 | −1.06 | −0.78 | −0.01 | 0.61 | |
| ANKRD22 | 4150270 | ILMN_7804 | 7 | 390 | −1.06 | −0.72 | −0.37 | 0.12 | |
| ERO1L | 4780671 | ILMN_4958 | 7 | 3416 | −1.05 | −1.04 | −0.61 | −1.02 | −0.69 |
| LOC650546 | 7210673 | ILMN_35170 | 7 | 1731 | −1.05 | −1.30 | −1.38 | −2.39 | |
| FCGR2A | 1990278 | ILMN_26366 | 7 | 558 | −1.04 | −1.16 | −1.19 | −1.14 | |
| HS.368984 | 5490192 | ILMN_87858 | 7 | 655 | −1.02 | −1.19 | −1.01 | −0.70 | |
| N4BP2L1 | 620112 | ILMN_24274 | 7 | 393 | −1.01 | −0.79 | −0.71 | −1.10 | −0.74 |
| MTUS1 | 3800017 | ILMN_5924 | 7 | 1134 | −1.00 | −1.12 | −1.51 | −1.69 | |
| SOX2 | 5080273 | ILMN_13292 | 7 | 622 | −1.00 | −1.22 | −1.22 | −1.35 | |
| KBTBD11 | 5050093 | ILMN_20625 | 7 | 5058 | −1.00 | −1.78 | −1.94 | −2.14 | −1.22 |

| | | | | | | | | | |
|---|---|---|---|---|---|---|---|---|---|
| NIN | 5310717 | ILMN_3063 | 7 | 4443 | −0.99 | −1.05 | −0.89 | −1.01 | −0.62 |
| ZBTB32 | 7560273 | ILMN_2617 | 7 | 2142 | −0.99 | −0.49 | −0.74 | −1.39 | |
| KLF9 | 3390292 | ILMN_2670 | 7 | 2736 | −0.98 | −1.40 | −1.07 | −1.08 | −1.14 |
| AQP9 | 160494 | ILMN_15164 | 7 | 13,906 | −0.97 | −1.31 | −1.44 | −1.95 | −1.63 |
| HS.580444 | 840452 | ILMN_132625 | 7 | 517 | −0.97 | −1.31 | −1.40 | −1.62 | |
| DMBX1 | 2940187 | ILMN_13566 | 7 | 1708 | −0.97 | −0.43 | −1.01 | −1.08 | −1.23 |
| MYO10 | 4670131 | ILMN_28857 | 7 | 621 | −0.97 | −1.13 | −0.79 | −0.76 | −1.70 |
| IKZF1 | 730482 | ILMN_22185 | 7 | 2966 | −0.95 | −0.65 | −0.36 | −1.26 | −0.68 |
| NIN | 2190196 | ILMN_3320 | 7 | 694 | −0.94 | −0.99 | −1.08 | −0.83 | |
| PLOD2 | 7040477 | ILMN_14675 | 7 | 14,800 | −0.94 | −0.81 | −0.66 | −1.06 | −0.78 |
| GPLD1 | 3180379 | ILMN_20927 | 7 | 557 | −0.93 | −0.87 | −1.00 | −0.84 | −0.81 |
| C13ORF18 | 7000079 | ILMN_19053 | 7 | 2630 | −0.92 | −1.57 | −1.57 | −1.99 | −1.19 |
| LOC647691 | 4920768 | ILMN_46634 | 7 | 1159 | −0.92 | −0.82 | −1.19 | −1.53 | |
| PRRX2 | 4150204 | ILMN_9456 | 7 | 3810 | −0.90 | −1.14 | −1.01 | −1.51 | −1.26 |
| NLRP7 | 5670129 | ILMN_2155 | 7 | 9786 | −0.89 | −0.45 | −0.68 | −1.02 | |
| HELLS | 7040161 | ILMN_164061 | 7 | 1893 | −0.89 | −0.82 | −1.44 | −1.92 | |
| KIAA1671 | 3120474 | ILMN_42090 | 7 | 2311 | −0.88 | −1.51 | −1.61 | −1.40 | |
| FLJ35880 | 1170241 | ILMN_3282 | 7 | 744 | −0.88 | −0.87 | −0.90 | −1.42 | |
| NLRP7 | 6180600 | ILMN_10029 | 7 | 3380 | −0.88 | −0.59 | −0.58 | −1.18 | |
| PLOD2 | 4640187 | ILMN_25982 | 7 | 2357 | −0.88 | −0.93 | −0.74 | −1.41 | |
| IRF4 | 6980370 | ILMN_12414 | 7 | 1389 | −0.88 | −0.81 | −1.48 | −1.81 | −1.67 |
| HS.205745 | 2640497 | ILMN_81914 | 7 | 713 | −0.87 | −1.11 | −0.44 | −0.34 | |
| DICER1 | 5390433 | ILMN_1996 | 7 | 1944 | −0.87 | −0.85 | −0.55 | −1.12 | −0.94 |
| HS.566469 | 1500133 | ILMN_118789 | 7 | 408 | −0.87 | −0.90 | −0.93 | −1.19 | |
| SLC19A3 | 4540593 | ILMN_25079 | 7 | 926 | −0.86 | −0.98 | −1.38 | −1.92 | −0.74 |
| TIPARP | 6760546 | ILMN_4419 | 7 | 10,212 | −0.86 | −1.44 | −1.69 | −2.14 | −1.13 |
| SF3B3 | 1090239 | ILMN_18316 | 7 | 9050 | −0.85 | −1.32 | −1.33 | −1.77 | |
| LOC442597 | 2710121 | ILMN_42881 | 7 | 69,455 | −0.85 | −1.33 | −1.26 | −1.51 | |
| CACNB2 | 7100154 | ILMN_2192 | 7 | 828 | −0.85 | −1.31 | −1.64 | −2.09 | −1.24 |
| SLC26A4 | 5900358 | ILMN_23896 | 7 | 5740 | −0.84 | −0.57 | −1.00 | −1.57 | −0.76 |
| IL1RAP | 3800427 | ILMN_8626 | 7 | 497 | −0.84 | −1.15 | −1.30 | −1.36 | −1.48 |
| CDK5RAP2 | 4260017 | ILMN_3896 | 7 | 26,180 | −0.83 | −0.98 | −1.23 | −1.07 | −1.48 |
| CSGALNACT2 | 1980092 | ILMN_11419 | 7 | 1414 | −0.83 | −1.02 | −0.80 | −1.11 | −1.26 |
| LOC650546 | 4180148 | ILMN_35170 | 7 | 2183 | −0.83 | −1.12 | −1.51 | −1.96 | |
| HS.566966 | 670411 | ILMN_119192 | 7 | 672 | −0.82 | −1.53 | −1.67 | −1.73 | |
| ZNF672 | 6330646 | ILMN_24640 | 7 | 4068 | −0.82 | −1.04 | −0.85 | −1.13 | |
| ZDHHC19 | 830615 | ILMN_27596 | 7 | 492 | −0.82 | −0.67 | −0.97 | −1.21 | −0.76 |
| SLCO2B1 | 1400121 | ILMN_1897 | 7 | 439 | −0.81 | −1.02 | −0.88 | −0.66 | −1.08 |
| C13ORF18 | 2850286 | ILMN_19053 | 7 | 3309 | −0.81 | −1.53 | −1.57 | −1.93 | −1.19 |
| ATF3 | 4780128 | ILMN_182693 | 7 | 2392 | −0.79 | −1.12 | −1.49 | −0.96 | |
| ITIH5 | 5050121 | ILMN_9175 | 7 | 442 | −0.78 | −0.85 | −1.03 | −1.10 | −0.95 |
| CSF3R | 6270114 | ILMN_21047 | 7 | 1542 | −0.78 | −1.42 | −1.04 | −0.86 | |
| CCDC34 | 3450408 | ILMN_2645 | 7 | 10256 | −0.77 | −0.79 | −1.19 | −1.24 | −1.55 |
| FAM123A | 2760291 | ILMN_26739 | 7 | 3304 | −0.76 | −1.21 | −1.55 | −2.32 | |
| HS.10862 | 1940563 | ILMN_71180 | 7 | 17,896 | −0.76 | −0.67 | −0.77 | −1.15 | |
| BATF3 | 5900253 | ILMN_7180 | 7 | 26,954 | −0.76 | −1.14 | −1.40 | −1.66 | −1.15 |
| ASPH | 2370450 | ILMN_182157 | 7 | 2107 | −0.76 | −1.01 | −1.49 | −1.51 | |
| TSHZ3 | 5870424 | ILMN_19320 | 7 | 8306 | −0.75 | −0.66 | −0.64 | −1.26 | −0.72 |
| RNF144 | 3450136 | ILMN_15740 | 7 | 3234 | −0.74 | −0.88 | −0.70 | −1.15 | |
| AGTPBP1 | 4760427 | ILMN_12151 | 7 | 2461 | −0.74 | −0.96 | −1.18 | −1.16 | −1.14 |
| BATF | 6220195 | ILMN_3307 | 7 | 6380 | −0.73 | −1.04 | −1.15 | −1.29 | |
| RUNX1 | 7400368 | ILMN_19125 | 7 | 1194 | −0.72 | −0.58 | −0.60 | −1.00 | |
| PCCA | 4730309 | ILMN_6045 | 7 | 3934 | −0.72 | −0.93 | −1.11 | −0.94 | |
| IL6R | 1780603 | ILMN_22419 | 7 | 329 | −0.70 | −0.96 | −0.84 | −1.00 | −0.61 |
| FAM113A | 290296 | ILMN_22766 | 7 | 2048 | −0.70 | −0.75 | −0.83 | −1.30 | |
| DDX12 | 2120039 | ILMN_33363 | 7 | 565 | −0.70 | −0.89 | −1.16 | −1.22 | |
| C1ORF112 | 940240 | ILMN_5134 | 7 | 2455 | −0.68 | −0.80 | −0.94 | −1.37 | −0.95 |
| MCM10 | 6580685 | ILMN_2483 | 7 | 1471 | −0.68 | −0.94 | −1.40 | −1.86 | −1.65 |
| PASK | 4150100 | ILMN_19873 | 7 | 1133 | −0.68 | −0.85 | −1.16 | −1.60 | −0.88 |
| TNFAIP3 | 3360681 | ILMN_2315 | 7 | 683 | −0.68 | −0.82 | −1.08 | −0.67 | |
| VCL | 2900068 | ILMN_26712 | 7 | 797 | −0.67 | −0.79 | −1.09 | −0.93 | |
| LOC653778 | 830639 | ILMN_32201 | 7 | 6816 | −0.67 | −0.77 | −0.66 | −1.39 | |
| CMTM7 | 2140239 | ILMN_8486 | 7 | 24,363 | −0.67 | −0.74 | −0.73 | −1.20 | −1.27 |
| SLC1A3 | 4210403 | ILMN_17250 | 7 | 494 | −0.67 | −1.21 | −0.92 | −1.45 | −0.77 |
| ACPP | 2680725 | ILMN_23186 | 7 | 836 | −0.66 | −1.03 | −0.83 | −0.73 | −0.75 |
| CACNB2 | 2850458 | ILMN_2192 | 7 | 930 | −0.66 | −1.09 | −1.32 | −1.94 | −1.24 |

| | | | | | | | | | |
|---|---|---|---|---|---|---|---|---|---|
| GNAL | 4200709 | ILMN_29214 | 7 | 918 | −0.65 | −0.72 | −1.17 | −1.17 | |
| CCDC34 | 7040184 | ILMN_2645 | 7 | 32,783 | −0.65 | −0.84 | −0.90 | −1.35 | −1.55 |
| NFKBIA | 4280113 | ILMN_6745 | 7 | 20,541 | −0.64 | −1.25 | −1.24 | −1.01 | −1.33 |
| LOC654103 | 7320707 | ILMN_37027 | 7 | 10,798 | −0.64 | −0.75 | −0.71 | −1.41 | |
| FAM29A | 4760615 | ILMN_3594 | 7 | 637 | −0.63 | −0.82 | −0.86 | −1.18 | −0.71 |
| HS.566835 | 4880398 | ILMN_119083 | 7 | 960 | −0.63 | −0.76 | −0.70 | −1.38 | |
| ACMSD | 20041 | ILMN_21475 | 7 | 1130 | −0.63 | −1.42 | −1.57 | −1.36 | |
| RAD54L | 1980291 | ILMN_1278 | 7 | 1080 | −0.63 | −1.04 | −1.26 | −1.69 | −1.71 |
| DPAGT1 | 2680176 | ILMN_10306 | 7 | 1281 | −0.63 | −0.49 | −0.76 | −1.09 | −0.65 |
| CLIP1 | 2260066 | ILMN_179647 | 7 | 8485 | −0.62 | −0.87 | −1.03 | −1.03 | |
| LOC653513 | 5820167 | ILMN_43919 | 7 | 1788 | −0.62 | −0.78 | −1.23 | −1.41 | |
| ARID2 | 7100044 | ILMN_163259 | 7 | 887 | −0.61 | −0.56 | −0.59 | −1.11 | |
| MAN1C1 | 6280184 | ILMN_12633 | 7 | 542 | −0.60 | −1.03 | −1.15 | −1.00 | |
| LOC440359 | 5870307 | ILMN_34340 | 7 | 10,799 | −0.60 | −0.67 | −1.02 | −1.50 | |
| HNRNPA3 | 1440592 | ILMN_5256 | 7 | 3138 | −0.59 | −1.09 | −1.17 | −1.63 | −0.66 |
| NOD1 | 7610390 | ILMN_10741 | 7 | 603 | −0.58 | −1.07 | −1.00 | −0.97 | −0.63 |
| NBL1 | 5090156 | ILMN_20462 | 7 | 6674 | −0.58 | −0.77 | −0.98 | −1.33 | |
| USP32 | 5290369 | ILMN_26659 | 7 | 1319 | −0.57 | −1.00 | −1.18 | −1.22 | −1.20 |
| MAPK14 | 4860600 | ILMN_17267 | 7 | 929 | −0.57 | −0.47 | −0.72 | −1.06 | −0.88 |
| HELLS | 4540768 | ILMN_3656 | 7 | 977 | −0.56 | −1.06 | −1.20 | −1.64 | −0.93 |
| GADD45B | 4920110 | ILMN_138334 | 7 | 1555 | −0.56 | −0.78 | −1.11 | −1.09 | |
| PIH1D1 | 5910202 | ILMN_10897 | 7 | 3009 | −0.56 | −0.74 | −0.90 | −1.04 | −0.88 |
| ARID5B | 1410408 | ILMN_17477 | 7 | 6846 | −0.56 | −0.88 | −1.21 | −1.48 | |
| ABHD3 | 5050241 | ILMN_4359 | 7 | 1158 | −0.56 | −0.73 | −0.59 | −1.20 | |
| FES | 4120689 | ILMN_27340 | 7 | 559 | −0.56 | −1.10 | −1.50 | −1.53 | |
| PCNA | 2510348 | ILMN_6858 | 7 | 579 | −0.56 | −0.85 | −0.99 | −1.08 | −0.81 |
| AK3L1 | 160148 | ILMN_28560 | 7 | 19,298 | −0.55 | −0.67 | −0.77 | −1.22 | |
| POLD3 | 4590608 | ILMN_24871 | 7 | 1295 | −0.54 | −1.04 | −1.04 | −1.63 | −0.95 |
| ANKRD32 | 6380747 | ILMN_15362 | 7 | 1552 | −0.54 | −0.67 | −0.57 | −1.07 | −0.60 |
| DDX23 | 5090450 | ILMN_2003 | 7 | 3819 | −0.53 | −0.52 | −0.83 | −1.05 | −0.67 |
| LOC654189 | 5700671 | ILMN_30702 | 7 | 995 | −0.52 | −0.80 | −0.62 | −1.30 | |
| CMTM7 | 4290403 | ILMN_8951 | 7 | 1952 | −0.52 | −0.82 | −0.92 | −1.36 | |
| SAPS3 | 2850273 | ILMN_6618 | 7 | 2917 | −0.51 | −1.06 | −1.01 | −0.79 | −0.73 |
| IMPA2 | 2340241 | ILMN_19881 | 7 | 6053 | −0.51 | −0.62 | −0.92 | −1.03 | −1.18 |
| MCC | 2480669 | ILMN_17732 | 7 | 434 | −0.50 | −0.89 | −0.84 | −1.02 | |
| HSD17B7 | 3420328 | ILMN_5318 | 7 | 30,246 | −0.50 | −0.71 | −0.68 | −1.09 | |
| TOPBP1 | 870601 | ILMN_24939 | 7 | 4008 | −0.49 | −0.90 | −1.00 | −1.22 | −0.72 |
| UCHL5IP | 1090370 | ILMN_27285 | 7 | 8570 | −0.49 | −1.15 | −1.34 | −1.78 | |
| PMP22 | 7560138 | ILMN_5694 | 7 | 14,585 | −0.48 | −0.64 | −0.53 | −1.02 | −0.71 |
| EME1 | 1260398 | ILMN_3574 | 7 | 1147 | −0.48 | −0.72 | −0.98 | −1.40 | −1.22 |
| EXO1 | 2690458 | ILMN_25997 | 7 | 707 | −0.48 | −1.00 | −1.28 | −1.36 | |
| CCDC4 | 5890554 | ILMN_1131 | 7 | 928 | −0.47 | −1.09 | −1.29 | −1.09 | |
| ZNF138 | 360370 | ILMN_36856 | 7 | 1182 | −0.46 | −0.71 | −0.67 | −1.23 | |
| DICER1 | 5130091 | ILMN_1996 | 7 | 8484 | −0.46 | −0.82 | −0.95 | −1.25 | −0.94 |
| HADH | 6290709 | ILMN_13258 | 7 | 8327 | −0.45 | −0.74 | −0.67 | −1.04 | −0.73 |
| TMEM106C | 1430537 | ILMN_7003 | 7 | 11,234 | −0.45 | −0.66 | −0.92 | −1.04 | −0.64 |
| PLOD2 | 460338 | ILMN_14675 | 7 | 612 | −0.44 | −0.84 | −0.56 | −1.18 | −0.78 |
| PDS5B | 5220575 | ILMN_13136 | 7 | 2131 | −0.44 | −0.64 | −0.73 | −1.03 | |
| PDS5B | 870167 | ILMN_20213 | 7 | 1213 | −0.42 | −0.78 | −0.92 | −1.20 | −0.78 |
| TNPO1 | 5080482 | ILMN_18758 | 7 | 5142 | −0.42 | −1.08 | −0.93 | −1.42 | |
| HS.135668 | 3830066 | ILMN_77684 | 7 | 436 | −0.41 | −0.86 | −0.70 | −1.01 | |
| CEP152 | 2340164 | ILMN_570 | 7 | 1068 | −0.41 | −0.84 | −0.94 | −1.39 | −1.47 |
| FLJ13305 | 5870072 | ILMN_5829 | 7 | 1312 | −0.39 | −0.62 | −0.94 | −1.08 | |
| FBXO5 | 5670255 | ILMN_9763 | 7 | 1243 | −0.39 | −1.22 | −1.35 | −1.46 | −1.46 |
| CHAF1A | 2120097 | ILMN_24131 | 7 | 775 | −0.38 | −0.87 | −1.02 | −1.32 | −0.97 |
| FANCD2 | 3610022 | ILMN_12871 | 7 | 451 | −0.38 | −0.73 | −1.02 | −1.06 | −0.93 |
| SPRYD5 | 4880204 | ILMN_14013 | 7 | 1048 | −0.37 | −1.04 | −1.30 | −1.18 | |
| DNA2 | 6380452 | ILMN_172479 | 7 | 872 | −0.37 | −0.69 | −0.90 | −1.13 | |
| SIN3A | 6420017 | ILMN_14108 | 7 | 4662 | −0.37 | −0.69 | −0.77 | −1.10 | −0.72 |
| TGIF2 | 1340020 | ILMN_25134 | 7 | 1540 | −0.37 | −0.71 | −0.47 | −1.00 | |
| ITK | 7560632 | ILMN_23317 | 7 | 1572 | −0.37 | −0.69 | −1.05 | −0.79 | |
| FAM80A | 6840619 | ILMN_12396 | 7 | 669 | −0.36 | −0.91 | −0.92 | −1.15 | |
| CDK2 | 2350286 | ILMN_12332 | 7 | 1377 | −0.35 | −0.90 | −0.93 | −1.14 | −1.13 |
| CACNB2 | 7210521 | ILMN_24772 | 7 | 326 | −0.35 | −0.50 | −0.68 | −1.00 | |
| RTTN | 6400632 | ILMN_5471 | 7 | 2581 | −0.34 | −0.88 | −0.81 | −1.10 | |
| PDK3 | 110347 | ILMN_15297 | 7 | 1622 | −0.33 | −0.67 | −0.78 | −1.07 | |

| | | | | | | | | | |
|---|---|---|---|---|---|---|---|---|---|
| CCNF | 3130541 | ILMN_27253 | 7 | 7467 | −0.31 | −0.78 | −0.80 | −1.21 | −0.96 |
| MTHFS | 6560066 | ILMN_1014 | 7 | 14,508 | −0.31 | −0.61 | −0.78 | −1.04 | −0.66 |
| SLC39A10 | 5960332 | ILMN_13415 | 7 | 1054 | −0.31 | −0.61 | −0.61 | −1.02 | |
| POLA2 | 4920537 | ILMN_8864 | 7 | 5588 | −0.30 | −0.58 | −0.71 | −1.01 | −0.82 |
| SLC37A4 | 5130577 | ILMN_24454 | 7 | 5049 | −0.28 | −0.87 | −0.82 | −1.25 | −0.84 |
| NUCKS1 | 1450753 | ILMN_17108 | 7 | 1290 | −0.25 | −0.97 | −0.87 | −1.38 | |
| MASTL | 580470 | ILMN_7073 | 7 | 957 | −0.23 | −0.92 | −0.84 | −1.06 | −1.06 |
| SHMT1 | 2690528 | ILMN_17710 | 7 | 3321 | −0.19 | −0.75 | −0.64 | −1.04 | −0.71 |
| FAM76B | 6130273 | ILMN_22478 | 7 | 1407 | −0.18 | −0.89 | −0.69 | −1.15 | |

AVG: Average.

**Table S2.** Cluster 7 genes selected for functional screening with shRNA.

| *Gene* | | *Cluster* | *Function* |
|---|---|---|---|
| **UPK1B** | Uroplakin 1B | 7 | Transmembrane 4,superfamily member (TM4SF) Mediatiates signal transduction events that play role in regulation of cell development, activation, growth and motility |
| **TNFRSF9** | Tumor necrosis factor receptor superfamily, member 9 (CD 137) | 7 | Plasma membrane receptor protein typical for TNFR superfamily. It contributes to the clonal expansion, survival, and development of T cells. |
| **TIPARP** | TCDD-inducible poly(ADP-ribose) polymerase | 7 | TIPARP alterates the function of target protein by trasfering ADP-ribose onto glutamic acid residues of a protein acceptor. It is associated to ovarian cancer. |
| **IRF4** | Interferon Regulator Factor 4 (MUM1) | 7 | It belongs to the IRF (interferon regulatory factor) family of transcription factors. IRF4 is often deregulated in Multiple Myeloma an Diffuse Large B Cell Lymphoma |
| **ITK** | IL2-inducible T cell Kinase | 7 | Is a member of BTK tyrosine kinase superfamily and plays an essential role in regulation of the development, function and differentiation of T and NKT cells |
| **ATF3** | Activating transcription factor 3 | 7 | Is a member of ATF/CREB family of transcription factors. ATF3 is induced by a variety of signals, including many of those encountered by cancer cells, and is involved in the complex process of cellular stress response |
| **BATF3** | Basic leucine zipper transcription factor, ATF-like 3 | 7 | This gene encodes a member of the basic leucine zipper protein family. The encoded protein functions as a transcriptional repressor when heterodimerizing with JUN. |
| **FGB** | Fibrinogen beta chain | 7 | This protein plays role in blood clotting and other responses to injury. Mutations in this gene lead to several disorders, including afibrinogenemia, dysfibrinogenemia. |
| **UHRF2** | Ubiquitin-like, containing PHD and RING finger domains | 7 | This protein mediates ubiquitination and subsequent proteasomal degradation. Important for G1/S transition. Overexpression causes G1 phase cell arrest. |
| **ICOS** | inducible T-cell co-stimulator | 7 | It s a major regulator of the adaptive immune system, required for effective T cell dependent immune responses |
| **MEIS2** | MEIS homeobox 2 | 7 | Is a member of TALE/MEIS homeobox family acting as a transcription repressor role in MLL leukemogenesis |
| **SOX2** | SRY (sex determining region Y)-box 2 | 7 | Transcription factor that controls the expression of a number of genes involved in embryonic development |
| **IL1RAP** | Interleukin 1 receptor accessory protein | 7 | This gene is a necessary part of the interleukin 1 receptor complex which initiates signaling events leading to the activation of interleukin 1-responsive genes |

**Table S3.** Schematic result of the functional shRNA screening on cluster 7 genes. Positive hits were selected according to the following criteria: more than one shRNA sequence was able to reduce target mRNA levels by at least 70%; there was a correlation between the proportion of gene silencing and the phenotype. For genes showing "strong" phenotype, a "rescue" experiment expressing a shRNA-resistant open reading frame was performed (ITK and IRF4 highlighted in black box).

| Gene | Phenotype | Rescue |
|---|---|---|
| UPK1B | No | |
| TNFRSF9 | No | |
| TIPARP | No | |
| IRF4 | Strong | Yes |
| ITK | Strong | No |
| ATF3 | Mild | |
| BATF3 | Mild | |
| FGB | No | |
| UHRF2 | No | |
| ICOS | No | |
| MEIS2 | No | |
| SOX2 | Mild | |
| IL1RAP | No | |

**Table S4.** List of shRNA sequences utilized in the screening. For each gene, 5 shRNA sequences from the TRC library (Sigma-Aldrich) were tested.

| Gene | shRNA sequences from A to E |
|---|---|
| SNFT | 3 A –ccgg-TGCTCAGAGAAGTCGGAAGAA-ctcgag-TTCTTCCGACTTCTCTGAGCA-tttt |
| | 3 B –ccgg-GCTGACAAGCTCCATGAGGAA-ctcgag-TTCCTCATGGAGCTTGTCAGC-tttt |
| | 3 C –ccgg-CCATGAGGAATATGAGAGCCT-ctcgag-AGGCTCTCATATTCCTCATGGT-tttt |
| | 3 D –ccgg-GCACCTGACAGAGGCACTGAA- ctcgag-TTCAGTGCCTCTGTCAGGTGCT-tttt |
| | 3 E –ccgg-CCCTATGAACTTTGTGCCAGT-ctcgag- ACTGGCACAAAGTTCATAGGGT-tttt |
| ATF3 | 12 A –ccgg-CCGCCTTTCATCTGGATTCTA-ctcgag-TAGAATCCAGATGAAAGGCGGT-tttt |
| | 12 E –ccgg-CCTCTTTATCCAACAGATAAA-ctcgag-TTTATCTGTTGGATAAAGAGGT-tttt |
| | 12 C –ccgg-CCTGAAGAAGATGAAAGGAAA-ctcgag-TTTCCTTTCATCTTCTTCAGGT-tttt |
| | 12 D –ccgg-GCTGAACTGAAGGCTCAGATT-ctcgag-AATCTGAGCCTTCAGTTCAGCT-tttt |
| | 12 B –ccgg-GCATTTGATATACATGCTCAA-ctcgag-TTGAGCATGTATATCAAATGCT-tttt |
| IRF4 | 45 A –ccgg-GCCCAAATTCTCCTCTCTAAA-ctcgag-TTTAGAGAGGAGAATTTGGGC-ttttt |
| | 45 B –ccgg-GCCATTCCTCTATTCAAGAAT-ctcgag-ATTCTTGAATAGAGGAATGGC-ttttt |
| | 45 C –ccgg-TGCGCTTTGAACAAGAGCAAT-ctcgag-ATTGCTCTTGTTCAAAGCGCAT-tttt |
| | 45 D –ccgg-CCAGCAGGTTCACAACTACAT-ctcgag-ATGTAGTTGTGAACCTGCTGGT-tttt |
| | 45 E –ccgg-GCTCTTTGACACACAGCAGTT-ctcgag-AACTGCTGTGTGTCAAAGAGCT-tttt |
| TNFRSF9 | 82 A –ccgg-CCGCAGATCATCTCCTTCTTT-ctcgag-AAAGAAGGAGATGATCTGCGGT-ttttg |
| | 82 B –ccgg-GCAGAAAGAAACTCCTGTATA-ctcgag-TATACAGGAGTTTCTTTCTGCT-ttttg |
| | 82 C –ccgg-GCAGGCAGTGTAAAGGTGTTT-ctcgag-AAACACCTTTACACTGCCTGCT-ttttg |
| | 82 D –ccgg-GCTGGTACATTCTGTGATAAT-ctcgag-ATTATCACAGAATGTACCAGCT-ttttg |
| | 82 E –ccgg-GCTCCGTTTCTCTGTTGTTAA-ctcgag-TTAACAACAGAGAAACGGAGCT-ttttg |
| TIPARP | 83 A –ccgg-GAAGGCAAGCTACTCTCATAA-ctcgag-TTATGAGAGTAGCTTGCCTTCT-tttt |
| | 83 B –ccgg-CCTTACTTACACTACTTACTT-ctcgag-AAGTAAGTAGTGTAAGTAAGGT-tttt |
| | 83 C –ccgg-GAGCAATGTGAGGATTCTATT-ctcgag-AATAGAATCCTCACATTGCTCT-ttttg |
| | 83 D –ccgg-AGGTCTTTGAGGCCAATATTA-ctcgag-TAATATTGGCCTCAAAGACCTT-ttttg |
| | 83 E –ccgg-TAGCAATGTCAACTCTATTTA-ctcgag-TAAATAGAGTTGACATTGCTAT-ttttg |
| ITK | 84 A –ccgg-TCAGTACACCAGTTCCACAGG-ctcgag-CCTGTGGAACTGGTGTACTGAT-tttt |
| | 84 B –ccgg-GCCTTATATGACTACCAAACC-ctcgag-GGTTTGGTAGTCATATAAGGCT-tttt |
| | 84 C –ccgg-CATCAACTATCACCAACATAA-ctcgag-TTATGTTGGTGATAGTTGATGTT-ttttg |
| | 84 D –ccgg-GTGAGAACAATCCCTGTATAA-ctcgag-TTATACAGGGATTGTTCTCACTT-ttttg |
| | 84 E –ccgg-GAAGACATCAGTACCGGATTT-ctcgag-AAATCCGGTACTGATGTCTTCTT-ttttg |

| | |
|---|---|
| **UPK1B** | 85 A –ccgg-GTAGCCTCAATTCTCCATTAA-ctcgag-TTAATGGAGAATTGAGGCTACTT-ttttg |
| | 85 B –ccgg-GAACCTGTGTTATCACAGTAA-ctcgag-TTACTGTGATAACACAGGTTCTT-ttttg |
| | 85 C –ccgg-CGGAGTGCATCTTCTTTGTAT-ctcgag-ATACAAAGAAGATGCACTCCGTT-ttttg |
| | 85 D –ccgg-CCTTCCAATGCTTCTGTTGAT-ctcgag-ATCAACAGAAGCATTGGAAGGTT-ttttg |
| | 85 E –ccgg-GCATAAAGTGTTGCCACCATA-ctcgag-TATGGTGGCAACACTTTATGCTT-ttttg |
| **FGB** | 67 A -ccgg-CTTCTGTATGTGACAACATTT-ctcgag-AAATGTTGTCACATACAGAAGT-ttttg |
| | 67 B -ccgg-GCACAGATGATGGTGTAGTAT-ctcgag-ATACTACACCATCATCTGTGCT-ttttg |
| | 67 C -ccgg-GATCCATATAAACAGGGATTT-ctcgag-AAATCCCTGTTTATATGGATCT-ttttg |
| | 67 D -ccgg-GCAACTAACCTTCGTGTGCTT-ctcgag-AAGCACACGAAGGTTAGTTGCT-ttttg |
| | 67 E -ccgg-CGTGTGCTTCGTTCAATCCTA-ctcgag-TAGGATTGAACGAAGCACACGT-ttttg |
| **UHRF2** | 71 A -ccgg-CGTCTCTTCTTCCATTACAAT-ctcgag-ATTGTAATGGAAGAAGAGACGT-tttt |
| | 71 B -ccgg-GAAGTTGTAAAGGCTGGTGAA-ctcgag-TTCACCAGCCTTTACAACTTCT-tttt |
| | 71 C -ccgg-ACTGGTATTGTCCTTCTTGTA-ctcgag-TACAAGAAGGACAATACCAGT-ttttt |
| | 71 D -ccgg-CTGCTGATGAAGACGTTATTT-ctcgag-AAATAACGTCTTCATCAGCAGT-tttt |
| | 71 E -ccgg-GTTGGTGATGTGGTAATGGTT-ctcgag-AACCATTACCACATCACCAAC-ttttt |
| **ICOS** | 13A -ccgg-GCATACTTATTTGTTGGCTTA-ctcgag-TAAGCCAACAAATAAGTATGCT-ttttg |
| | 13B -ccgg-CCTTTGTTGTAGTCTGCATTT-ctcgag-AAATGCAGACTACAACAAAGGT-ttttg |
| | 13C -ccgg-CTGCCAATTATGAGATGTTTA-ctcgag-TAAACATCTCATAATTGGCAGT-ttttg |
| | 13D -ccgg-CCATTCTCATGCCAACTATTA-ctcgag-TAATAGTTGGCATGAGAATGGT-ttttg |
| | 13E -ccgg-GCTGAAGTTCTGGTTACCCAT-ctcgag-ATGGGTAACCAGAACTTCAGCT-ttttg |
| **MEIS2** | 69 A -ccgg-CGGCCTTTGTTCCTCCATAAA-ctcgag-TTTATGGAGGAACAAAGGCCGT-tttt |
| | 69 B -ccgg-CCCATGATTGACCAGTCAAAT-ctcgag-ATTTGACTGGTCAATCATGGGT-tttt |
| | 69 C -ccgg-CCACCGATACATTAGCTGTTT-ctcgag-AAACAGCTAATGTATCGGTGGT-tttt |
| | 69 D -ccgg-CCACAAATCTCGCTGACCATA-ctcgag-TATGGTCAGCGAGATTTGTGGT-tttt |
| | 69 E -ccgg-CCAAGTAAACAACTGGTTTAT-ctcgag-ATAAACCAGTTGTTTACTTGGT-tttt |
| **SOX2** | 70 A -ccgg-GAAGAAGGATAAGTACACGCT-ctcgag-AGCGTGTACTTATCCTTCTTCT-tttt |
| | 70 B -ccgg-CTGCCGAGAATCCATGTATAT-ctcgag-ATATACATGGATTCTCGGCAGT-tttt |
| | 70 C -ccgg-CAGCTCGCAGACCTACATGAA-ctcgag-TTCATGTAGGTCTGCGAGCTGT-tttt |
| | 70D -ccgg-TGGACAGTTACGCGCACATGA-ctcgag-TCATGTGCGCGTAACTGTCCAT-ttttg |
| | 70E - ccgg-CAACGGCAGCTACAGCATGAT-ctcgag-ATCATGCTGTAGCTGCCGTTGT-ttttg |
| **IL1RAP** | 27A -ccgg-CGCATTAGTAAGGAGAAAGAT-ctcgag-ATCTTTCTCCTTACTAATGCGT-ttttg |
| | 27B -ccgg-CCTCTCGTATTCATCTTTGAA-ctcgag-TTCAAAGATGAATACGAGAGGT-ttttg |
| | 27C -ccgg-CCCAGTGCATAAACTGTATAT-ctcgag-ATATACAGTTTATGCACTGGGT-ttttg |
| | 27D -ccgg-TCATTCCCTGTACGGTCTATT-ctcgag-AATAGACCGTACAGGGAATGAT-ttttg |
| | 27E -ccgg-CCATGTTTACTGGCTAGAGAT-ctcgag-ATCTCTAGCCAGTAAACATGGT-ttttg |

**Table S5.** Primer sequences used in the present study.

| Gene Name | Forward Primers | Reverse Primers |
|---|---|---|
| TPARP | 5′-CCACACCACCCTCTAGCAAT-3′ | 5′-CTTTCAGACCCCGAGAGTTG-3′ |
| ITK | 5′-CGATTCCATCCCTCTTCTCA-3′ | 5′-TTCTGCCTCCCAAAACAAAC-3′ |
| TNFRSF9 | 5′-CACTCTGTTGCTGGTCCTCA-3′ | 5′-GCGCTGGAGAAACTATTTGG-3′ |
| UPK1B | 5′-CCCTCCAAACAATGATGACC-3′ | 5′-CCTCTGCCGGAGAAACAG-3′ |
| ATF3 | 5′-AAGGAAAAAGAGGCGACGAG-3′ | 5′-GCTGCTTCTCGTTCTTGAGC-3′ |
| SNFT | 5′-AGCCCTGAGGATGATGACAG-3′ | 5′-GTTTTCTTGCTCCAGGCTCTC-3′ |
| IRF4 | 5′-ACCCCTACACCATGACAACG-3′ | 5′-GTCCAAACGTCATGGGACAT-3′ |
| SOX2 | 5′-CTACGACGTGAGCGCCCTGC-3′ | 5′-GCCAAGAGCCATGCCAGGGG-3′ |
| UHRF2 | 5′-ACTGGCTGGTGGATTTGCGGA-3′ | 5′-CCAGTCTTCCACCCGGTATCTGGA-3′ |
| FGB | 5′-GGGCTCGTCCAGCCAAAGCA-3′ | 5′-ACACCCCCAGGTCTGGGTCA-3′ |
| MEIS2 | 5′-GCCAAACGTCCCCGGCAAGA-3′ | 5′-AGCGGGAACCCCTACTCCGT-3′ |
| ICOS | 5′-GTGCTCACTGGGAGTGGAAT-3′ | 5′-GTCAACTGGGGTTCAGCAAT-3′ |
| Il1RAP | 5′-TGGTTGTTCTAAGCCCCAAC-3′ | 5′-ACCTGCCCTGTGGATACTTG-3′ |
| STAT3 | 5′-CTGCTCCAGGTACCGTGTGT-3′ | 5′-CCTCTGCCGGAGAAACAG-3′ |
| TNFRSF8 | 5′-GTCACCCACCTGACATCACC-3′ | 5′-CCATGACATCCACTGTTCCA-3′ |
| GAPDH | 5′-AAGGAGAGCTCAAGGTCAGC-3′ | 5′-GGGAGTAGGGACCTCCTGTT-3′ |

© 2018 by the authors; licensee MDPI, Basel, Switzerland. This article is an open access article distributed under the terms and conditions of the Creative Commons by Attribution (CC-BY) license (http://creativecommons.org/licenses/by/4.0/)
